# A Rapid Antibody Enhancement Platform in *Saccharomyces cerevisiae* Using an Improved, Diversifying CRISPR Base Editor

**DOI:** 10.1021/acssynbio.3c00299

**Published:** 2023-10-24

**Authors:** Andrew P. Cazier, Olivia M. Irvin, Lizmarie S. Chávez, Saachi Dalvi, Hannah Abraham, Nevinka Wickramanayake, Sreenivas Yellayi, John Blazeck

**Affiliations:** † School of Chemical and Biomolecular Engineering, Georgia Institute of Technology, Atlanta, Georgia 30332, United States

**Keywords:** CRISPR, base editing, yeast display, antibody, directed evolution, synthetic biology

## Abstract

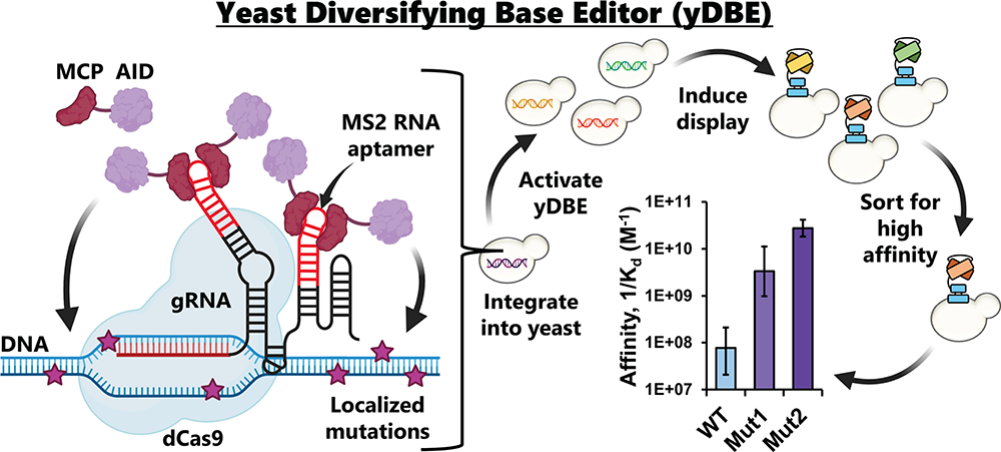

The yeast *Saccharomyces cerevisiae* is commonly used to interrogate and screen protein variants and to perform directed evolution studies to develop proteins with enhanced features. While several techniques have been described that help enable the use of yeast for directed evolution, there remains a need to increase their speed and ease of use. Here we present yDBE, a yeast diversifying base editor that functions *in vivo* and employs a CRISPR-dCas9-directed cytidine deaminase base editor to diversify DNA in a targeted, rapid, and high-breadth manner. To develop yDBE, we enhanced the mutation rate of an initial base editor by employing improved deaminase variants and characterizing several scaffolded guide constructs. We then demonstrate the ability of the yDBE platform to improve the affinity of a displayed antibody scFv, rapidly generating diversified libraries and isolating improved binders via cell sorting. By performing high-throughput sequencing analysis of the high-activity yDBE, we show that it enables a mutation rate of 2.13 × 10^–4^ substitutions/bp/generation over a window of 100 bp. As yDBE functions entirely *in vivo* and can be easily programmed to diversify nearly any such window of DNA, we posit that it can be a powerful tool for facilitating a variety of directed evolution experiments.

## Introduction

Directed evolution via DNA mutagenesis and screening of the resultant protein libraries is an essential strategy for improving protein function.^1^ The yeast *Saccharomyces cerevisiae* is frequently used for directed evolution experiments because it grows rapidly, has well developed genetic tools, can carry out eukaryotic posttranslational modifications, and can be engineered to display proteins or protein fragments tethered to the cell surface.^2−5^ The majority of directed evolution programs in yeast utilize mutant libraries created through traditional, *in vitro* mutagenesis techniques such as site-saturation mutagenesis or error-prone PCR.^6−9^ While these methods introduce sufficient diversity, they require laborious *in vitro* cloning procedures that slow the iterative process of directed evolution.

To circumvent these issues, a number of methods have been developed to continuously generate genetic diversity within a cell.^10,11^ Within yeast specifically, tet-directed DNA glycosylases (TaGTEAM),^12^ CRISPR-targeted error-prone DNA polymerases (EvolvR),^13^ a T7-polymerase-guided cytidine deaminase (TRIDENT),^14^ retrotransposon cycling with an error-prone reverse transcription step (ICE),^15^ and error-prone orthogonal DNA polymerases contained on cytoplasmic plasmids (OrthoRep and AHEAD)^16,17^ enable DNA mutation *in vivo*. These systems, with the exception of EvolvR, are unable to target multiple sequence regions and require a targeted gene to be first inserted in a predefined location. The rate of DNA diversification, as measured by substitutions per base pair per generation (s.p.b.), can vary widely in these systems. As an example, OrthoRep, which reports a mutation rate of 1 × 10^–5^ s.p.b., in some cases took up to 13 passages, or up to 90 generations, to evolve a desired resistance phenotype.^16^ The highest reported mutation rate attained by these yeast-based systems is 1 × 10^–3^ s.p.b. using TRIDENT.^14^

CRISPR base editors can mediate *in situ* DNA mutation in a targeted manner by employing programmable DNA binding proteins, such as dCas9, to target cytidine or adenine deaminases to specific DNA sequences.^18^ In this way, nucleotide deaminases are directed to a specific locus that bears homology to a 20-bp spacer sequence within a CRISPR guide RNA (gRNA), resulting in DNA mutations near the targeted site. The deaminase can be fused directly to the dCas9 protein or recruited through a secondary protein–protein or protein–RNA interaction, for instance, by incorporating MS2 aptamer sequences into a gRNA scaffold and taking advantage of the high affinity interaction between the MS2 phage coat protein (MCP) and MS2 aptamers.^19−21^ In yeast specifically, CRISPR base editors have been employed to perturb essential genes using dCas9 fusions of *Petromyzon marinus* CDA1 or human APOBEC3A deaminases.^22,23^ Cytidine deaminases transform cytosine to uracil in ssDNA. Uracil is recognized as DNA damage, but it is often repaired inaccurately, leading to permanent DNA mutations.^24^ Possible outcomes of cytosine deamination to uracil include (1) replacement with thymine as the DNA undergoes replication, (2) excision and replacement with any nucleotide through base excision repair, or (3) mismatch repair, especially when one strand of DNA is nicked, causing mutations at or near the uracil.^25,26^ In this way, a variety of substitutions can occur at or near the deaminated cytosine.

Many CRISPR base editor engineering efforts have increased the precision and specificity of DNA mutagenesis to enable their application in gene editing technologies, for instance, for clinical application in which only one specific DNA base pair mutation is desired or allowable.^27−30^ To give two specific examples, a uracil glycosylase inhibitor domain or a uracil DNA glycosylase can be incorporated into a base editor to help reinforce C-to-T or C-to-G mutations, respectively.^27,31^ In contrast, diversifying base editors (DBEs), are designed to generate a high mutational load via a variety of substitutions in the vicinity of their target site with applications in directed evolution.^25,32,33^ For instance, the CRISPR-X technique utilized a human activation-induced cytidine deaminase (AID)-MCP fusion protein to mutate DNA in mammalian cells^34^ and recapitulate aspects of antibody affinity maturation.^35^ AID is the catalytic deaminase enzyme that mediates somatic hypermutation of antibody sequences, i.e., their mutation, in B cells.^36^

Antibody therapeutics have seen tremendous growth over the past decade and are used to treat a variety of diseases, including viral infections, autoimmune disorders, and cancer.^37^ Due to its ability to grow rapidly to high densities and surface-present libraries of antibody variants, *S. cerevisiae* has become a popular platform for therapeutic antibody interrogation. A recently described *in vivo* continuous evolution platform for the isolation of high affinity nanobodies in yeast, AHEAD, demonstrates the remarkable potential of combining *in situ* DNA diversification with yeast protein display.^17^ Surprisingly, to the best of our knowledge, DBEs have not been designed or employed for use in yeast despite the potential applications of such a system.

In this work, we created and then improved a yeast DBE (yDBE) and established that it effectively mediated the targeted DNA diversification of both an enzyme and an antibody fragment. Using a fluorescence shift assay of GFP enzyme variants, we improved the initial mutagenesis capability of our yDBE by (1) identifying a highly active AID variant from a panel of previously described or novel AID upmutants,^38^ (2) adjusting the number and placement of MS2 aptamers housed within the gRNA scaffold to find complementary scaffolds with high activity and unique targeting profiles, and (3) increasing the versatility of the yDBE platform to promote multiloci targeting using rapidly assembled, tRNA-gRNA cassettes. We then demonstrated that the yDBE platform could be utilized to improve the affinity of an antifluorescein scFv by over 100-fold through *in situ* DNA diversification coupled with yeast display. This work demonstrates the first development of a diversifying base-editor system for targeted and rapid DNA diversification in yeast. Furthermore, our work is the first instance in which the human AID enzyme has been employed for CRISPR base editing in yeast. Lastly, yDBE enables a mutation rate of 2.13 × 10^–4^ s.p.b. over a window of 100 bp, approaching prior best-in-class *in vivo* mutagenesis studies.

## Results and Discussion

### Development of an Initial CRISPR Diversifying Base Editor for Yeast (yDBE)

**Figure 1:**
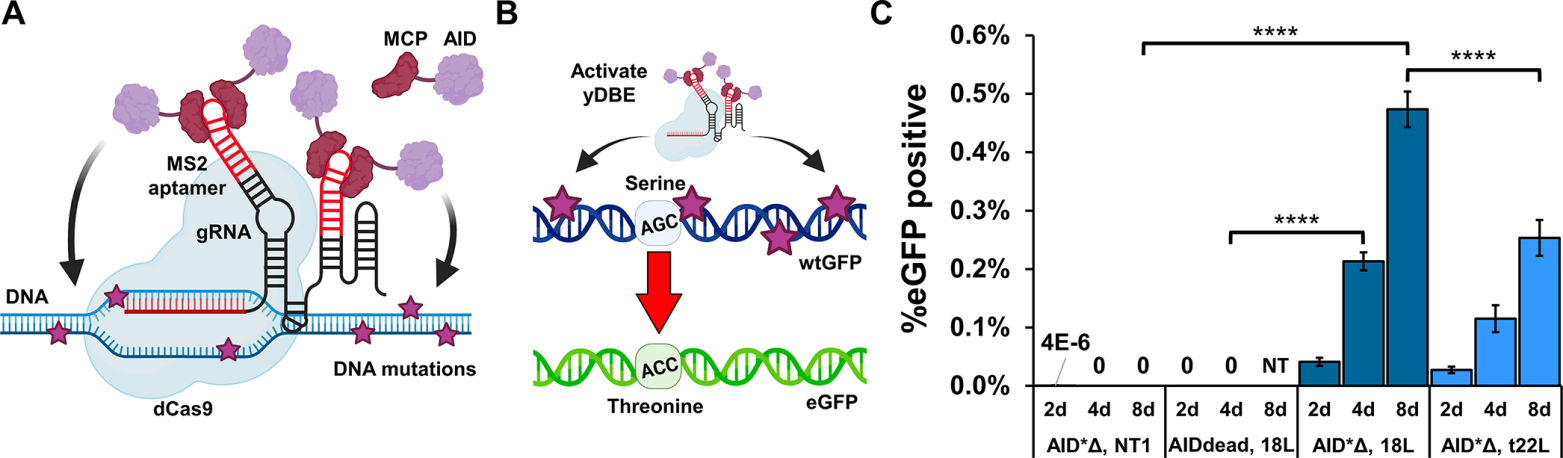
Development of an initial CRISPR diversifying base editor for yeast (yDBE). (A) A diagram of yDBE. MCP-AID, dCas9, and an MS2-loop-harboring gRNA together induce mutations near the targeted locus. (B) Outline of fluorescence shift assay for detecting DNA mutations induced by the base editor. The S65T mutation in wild-type GFP (wtGFP) shifts the fluorescence excitation peak, yielding fluorescence-like enhanced GFP (eGFP). (C) Fluorescence shift assay results with the initial base editor (strain AC001) and a nontargeting gRNA (NT1) or one of two targeting gRNAs (18L and t22L), all with M13 scaffolds. In C, bars represent mean ± SD, *n* = 3, ****p < 0.0001. NT, not tested.

We engineered a programmable, yeast-based diversifying base editor strain for preliminary testing by genomically integrating codon-optimized MCP-AID*Δ and dCas9 proteins. When coupled with a gRNA encoding MS2 aptamer loops, we expected that our yDBE could enable targeted DNA mutation in yeast (Figure 1A), mimicking the capabilities and design of the CRISPR-X platform developed in mammalian cells.^34^ MCP forms dimers when binding MS2 aptamers, allowing multiple AID*Δ proteins to be recruited to the targeted site. AID*Δ is a more active mutant of human activation-induced cytidine deaminase (Supplemental Table S1). dCas9 and MCP-AID*Δ were placed under the control of galactose-inducible promoters, pGAL1 and pGAL2, respectively. Wild-type GFP (wtGFP) was also genomically integrated at a separate locus with the constitutive Ptdh3 promoter driving its expression. We utilized integration sites (YORWΔ22 and YPRCτ3) that are known to afford robust transgene expression.^39^ Lastly, scaffolded gRNAs were expressed on a plasmid that was transformed into the yeast after the base editor integration. Our initial system used gRNAs with an M13 scaffold in which two MS2 loops were incorporated in the scaffold in the first and third loops of the gRNA as will be described further below.

We then used a fluorescence shift-based assay to determine if our initial yDBE platform could introduce targeted mutations into the wtGFP enzyme.^34^ Compared to wtGFP, enhanced GFP (eGFP) has an S65T mutation that shifts the excitation spectra peak from 405 to 488 nm (Figure 1B).^40^ AID naturally prefers to deaminate cytidines within a WRCY nucleotide motif, especially the palindromic AGCT.^36^ As the S65 amino acid in wtGFP is part of an AGCT nucleotide motif, targeting this region with an AID-based base editor can result in wtGFP → eGFP mutations over time, allowing sensitive detection of base editor activity via flow cytometry and the use of fluorescence shift percentage as a correlate for the overall base editor mutation rate (Supplemental Figure S1). Using our initial yDBE system targeted by either of two different M13 scaffolded gRNAs, we were able to generate a small fraction of yeast cells, <0.05%, that displayed eGFP’s excitation after 2 days induction (Figure 1C). Furthermore, we sequenced DNA from cells within the eGFP population to verify the presence of the expected S65T mutation, confirming the yDBE function. We measured the fluorescence at 2, 4, and 8 days and observed that mutations accumulated roughly linearly over time (Figure 1C), approaching 0.5% eGFP+ cells. GFP-targeting gRNAs are named as follows: nucleotide distance from the S65 target site to the 3′ end of the gRNA PAM (NGG-3′), L or R for left or right in the direction of the GFP open reading frame, and either a “t” for gRNAs that target dCas9 to bind to the template strand or no additional symbol for the coding strand.

As a prior study revealed that dCas9 alone can result in mutations in a targeted locus,^41^ we verified that AID was required to induce the S65T mutation by determining that a modified yDBE strain harboring a catalytically dead mutant (AIDdead) did not result in any eGFP+ cells after an 4-day induction (Figure 1C). Similarly, the initial yDBE did not result in accumulation of eGFP+ cells when used with a nontargeting gRNA, NT1 (Figure 1C).

### Employing Higher Activity AID Variants to Enhance yDBE Mutation Rate

After confirming the functionality of a yDBE, we sought to improve its activity through two main strategies: (1) improving the activity or expression of the deaminase and (2) optimizing the location of the MS2 aptamers within the gRNA scaffold. In our first strategy, we began by exploring several methods to increase the expression of AID*Δ and, thereby, increase base editing activity. We found that (1) altering the codon optimization,^34,42^ (2) changing the GAL2 promoter to a strong, orthologous, constitutive promoter, or (3) using an altered MCP variant^43^ either did not change or decreased the activity relative to the initial base editor (Supplemental Figure S2A). Next, we made base editor variants that included yeast ssDNA binding protein RFA3, a subunit of the replication factor A (RPA) complex. Fusing RFA3 to rat APOBEC1 (a cytidine deaminase related to AID) has been shown to increase the rate of genome-wide mutations.^44^ We fused RFA3 to the C-terminus of MCP-AID*Δ or directly to dCas9 and performed fluorescence shift assays but again did not detect an increase in eGFP mutations (Supplemental Figure S2B and S2C). Finally, we also confirmed that employing MS2 aptamer scaffolded gRNAs to recruit MCP-AID to a dCas9-targeted DNA locus outperforms a direct dCas9-AID fusion protein in our fluorescence shift assay (Supplemental Figure S2D). While it is possible that the gRNA we used for this comparison favored the MS2-based system, previous work in mammalian cells has also shown that base editors that use MCP-MS2 recruitment induce broader mutations compared to direct dCas9-deaminase fusions.^19^ Therefore, we pursued additional strategies to enhance the MS2-based system.

**Figure 2:**
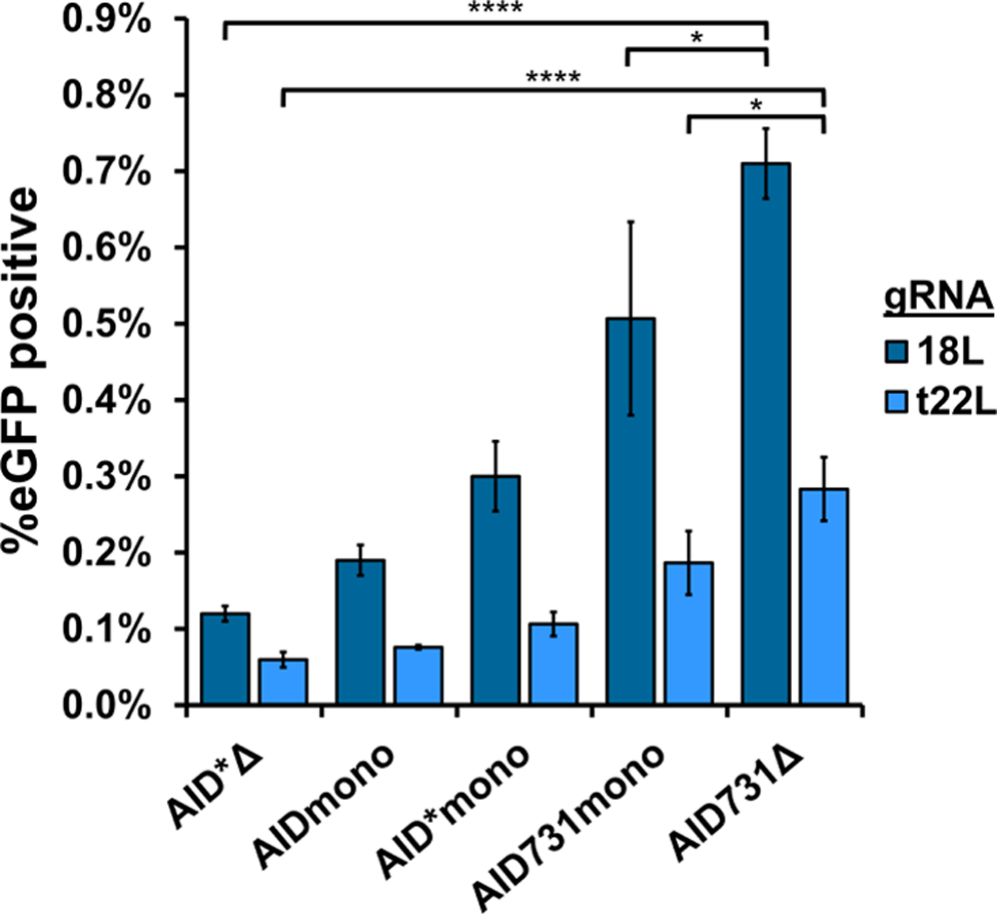
Employing higher activity AID variants to enhance yDBE mutation rate. Mutants of AID fused to MCP were tested in a fluorescence shift assay with two targeting gRNAs (18L and t22L) with an M13 scaffold. Cells were induced for 24 h. Bars represent mean ± SD, *n* = 3, *p < 0.05 and ****p < 0.0001.

As attempts to alter expression or fuse mutation-enhancing factors to yDBE failed to noticeably improve its function, we next investigated if engineered AID variants with higher catalytic activity might improve yDBE-mediated mutation rates. AID*Δ has a premature stop codon (195*) to remove its final three residues that can mediate nuclear export.^34,45^ All of our tested variants also lack this nuclear export signal. AID*Δ, which contains three coding mutations, K10E, T82I, and E156G, was isolated from previous work that measured global mutation rates of AID variants in *E. coli*.^38^ Interestingly, a related variant was reported to have more than 5-fold activity relative to the K10E, T82I, and E156G mutant. This variant, referred to as “Mut7.3.1,” contains 9 coding mutations, including the 3 coding mutations found in AID*Δ, though it still retained a nuclear export signal (Supplemental Table S1). Separate AID engineering efforts have also generated a variant dubbed AIDmono that showed higher activity as a base editor.^19,46^ To determine if use of an enhanced AID variant could improve the mutational rate, we fused the Mut7.3.1 (AID731Δ) and AIDmono variants to MCP, as well as combined mutations from AID*Δ, Mut7.3.1, and AIDmono into novel variants: AID*mono and AID731mono (Supplemental Table S1). We tested their activity in comparison to our initial yDBE in the context of two targeting (18L, and t22L) gRNAs (Figure 2). Promisingly, the best performing variant, AID731Δ, had at least a 5-fold increase in activity in mutation rate, as assessed by the eGFP fluorescence shift, relative to AID*Δ. The improvement was consistent for both the 18L and t22L gRNA targeting sequences, which target different DNA strands (coding versus template) at differential distances from the nucleotides that encode the initial S65 residue. After only 1 day of yDBE induction with the 18L gRNA, 0.71% of cells harboring the AID731Δ yDBE were positive for the eGFP mutation compared to 0.12% of AID*Δ cells.

### Varying MS2 Aptamer Placement

As a second strategy to increase the yDBE mutational rate, we sought to determine the ideal location and number of MS2 aptamers within the gRNA framework. Previous studies have characterized the impact of different MS2 aptamer locations in gRNAs, typically in mammalian cell hosts.^20,43,47,48^ As the effect of MS2 aptamer placement within gRNAs has not been characterized previously in yeast or in the context of DBEs, we analyzed a comprehensive set of gRNA/MS2 aptamer designs using our initial yDBE that employed AID*Δ.

**Figure 3:**
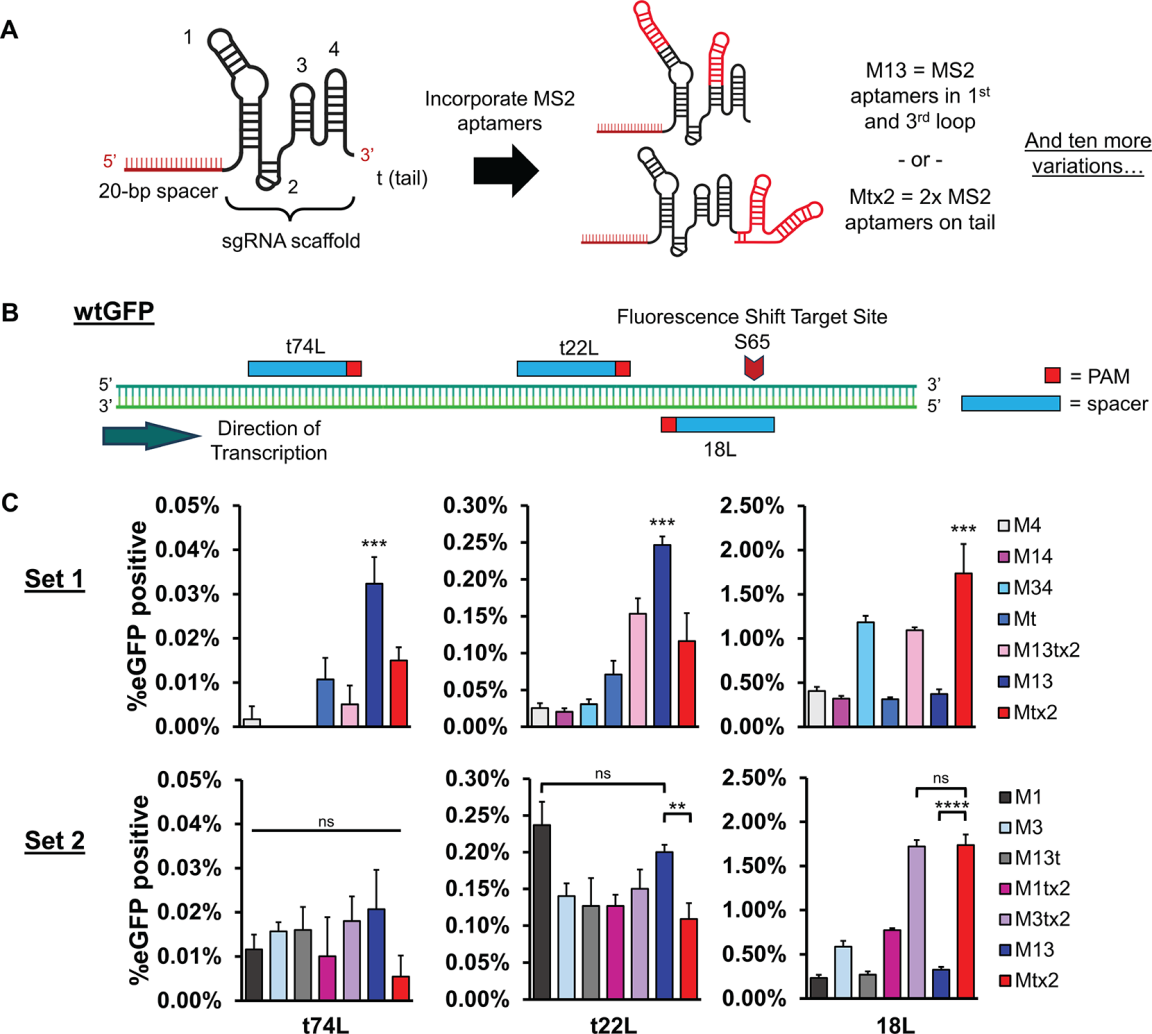
Varying MS2 aptamer placement to increase diversification. (A) Diagram of gRNA scaffold base pairing and possible positions for MS2 loop placement. Two examples drawn with MS2 loops are shown, M13 and Mtx2, but ten more variants were also constructed and tested. (B) Gene map showing the relative position of the S65 target site within wtGFP and the targeting location of three gRNA spacer sequences, 18L, t22L, and t74L. (C) Comparison of fluorescence shift assay activity using MCP-AID*Δ (strain AC001) and different gRNA scaffolds. Cells were induced for 4 days. Two separate sets of scaffolds were tested, each with three different spacer sequences. M13 and Mtx2 were assayed in both sets, so that a comparison between sets could be made. In C, bars represent mean ± SD, *n* = 3, ns = not significant, **p < 0.01, ***p < 0.001, ****p < 0.0001.

gRNAs that complex with the SpCas9 protein naturally contain four loops (Figure 3A), of which three (loops 1, 3, and 4) support insertion of an MS2 aptamer sequence, i.e., an MS2 loop. MS2 loops can also be appended to the “tail” or 3′ end of the gRNA. In our nomenclature, MS2 loop insertion into natural gRNA loops is denoted by the loop number, while inclusion on the gRNA tail is denoted by a “t”. A tandem repeat of the MS2 loop on the gRNA tail, denoted with “tx2”, has been described previously and was also tested by itself and in combination with other MS2 insertions.^43^

In total, we constructed and tested 11 different scaffolded gRNA constructs, dubbed M1, M3, M4, M14, M34, Mt, Mtx2, M1tx2, M3tx2, M13t, and M13tx2, and compared them to our starting configuration, M13. Across two experiments, we assessed the mutational capacity bestowed by each MS2 loop configuration in the yDBE system using fluorescence shift assays, and we tested each MS2 loop configuration in the context of three gRNA spacer sequences to ensure effects were not gRNA dependent (Figure 3B, C). Two configurations, M13 and Mtx2, were repeated in both experiments to allow a qualitative comparison between the sets. The M13 and Mtx2 configurations were selected for further analysis due to their superior performance with the t22L/t74L and 18L gRNAs, respectively. While M3tx2 had a signal comparable to Mtx2, additional tests with alternative spacer sequences did not demonstrate significant improvement (Supplemental Figure S3). The Mtx2 configuration and 18L gRNA combination improved EGFP+ mutation occurrence 5-fold compared to our initial M13 configuration used in yDBE.

**Figure 4:**
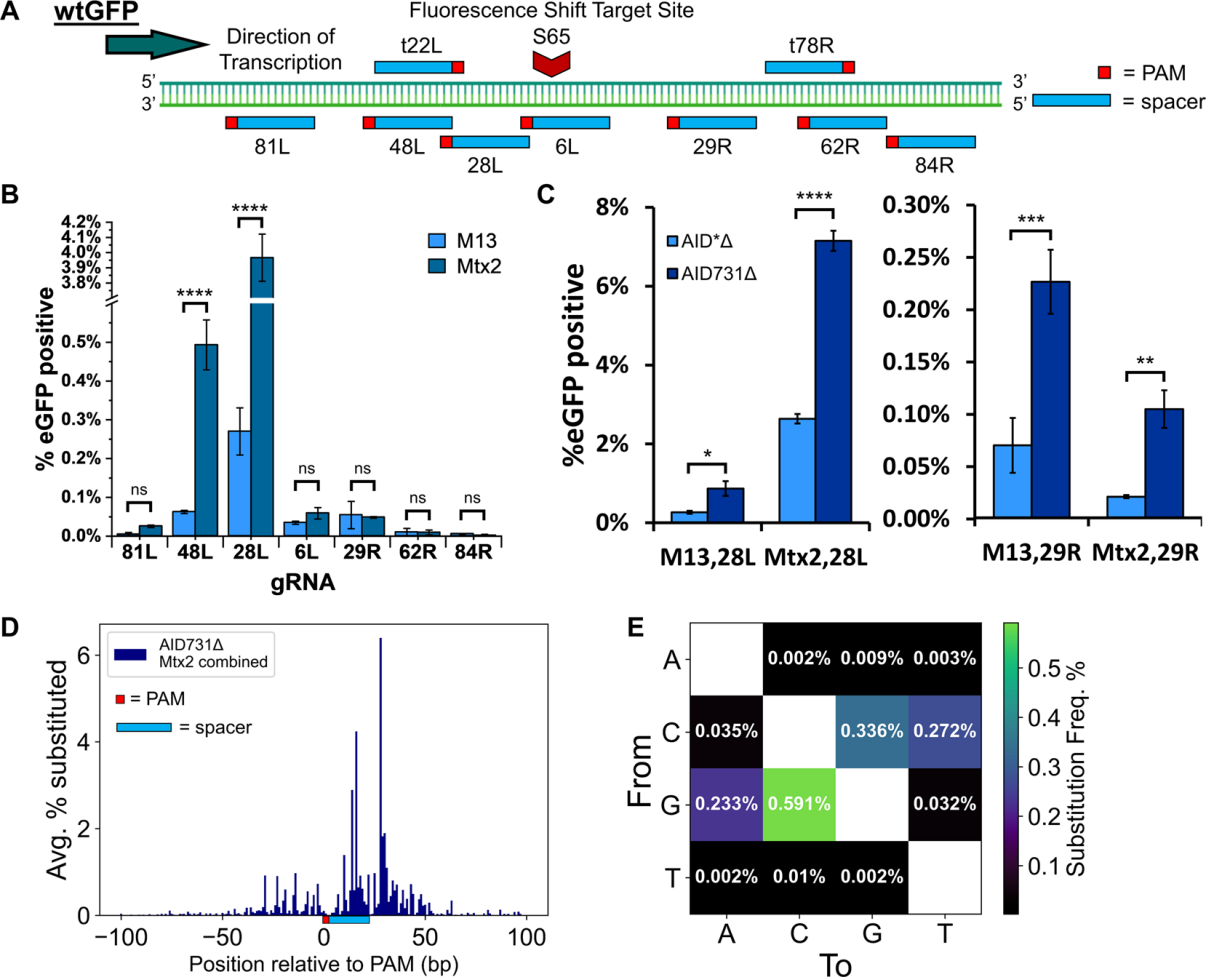
Target site tiling and high-throughput sequencing of yDBE. (A) Position of spacer sequences relative to fluorescence shift site (S65). The spacer number is a measure of how many bp the 3′ end of the PAM is to the left (L) or right (R) of the target on the coding or template (t) strand. (B) Comparison of M13 and Mtx2 scaffolds with seven positional spacers in a fluorescence shift assay in strain AC001. Cells were induced for 4 days. (C) Comparison of M13 and Mtx2 scaffolds in a fluorescent shift assay with AID731Δ (strain AC003) or the original AID*Δ (strain AC001). Cells were induced for 4 days. (D) Combined high-throughput sequencing results using AID731Δ (strain AC003) and Mtx2 scaffold with five separate spacer sequences, 81L, t22L, 28L, 29R, and t78R. The cells were induced for 8 days. The plot gives the average substitution frequencies for each nucleotide position relative to the 3′ end of the PAM in a ±100-bp window surrounding the targeted site. The orientation and binding position of the gRNA are depicted along the *x*-axis. The uncombined data is given in Supplemental Figure S4. (E) Heatmap detailing the nature of substitutions in a ±50-bp window centered on the PAM using the data highlighted in D. In B and C, bars represent mean ± SD, *n* = 3, ns = not significant, *p < 0.05, **p < 0.01, ***p < 0.001, ****p < 0.0001.

We next wanted to understand the mutagenic window afforded by the M13 and Mtx2 loop configurations, as a larger nucleotide range in which mutations occur is beneficial for a DBE. Previous work in mammalian cells showed that while mutations could be detected −50 to +50 bp relative to the CRISPR PAM and the direction of transcription, the highest rate of mutation was seen from +20 to +40 bp,^34^ independent of the DNA strand being targeted. We approximated the mutational window allowed by the M13 and Mtx2 loop configurations by using seven distinct gRNAs targeted (i.e., were complementary to) across the breadth of the coding strand of wtGFP (Figure 4A). The 3′ end of the PAMs of the gRNA spacer sequences ranged from −81 bp (Left, or L) to +84 bp (Right, or R) relative to the site of the desired mutation at S65T. We found that for both the M13 and Mtx2 scaffolds, the mutation rate was highest when using the 28L gRNA (Figure 4B), and that as expected, mutation rates reduced substantially for the most distant gRNAs. As the Mtx2 scaffolded design significantly outperformed M13 when aimed to the left of the target site, affording by far the highest mutation rates detected, we proceeded to further characterize the combination of the AID731Δ variant and the Mtx2 scaffold.

### Combining AID731Δ and Mtx2 Scaffold

By combining the Mtx2 scaffold with AID731Δ, we achieved a rate of wtGFP to eGFP fluorescence shift of over 7% with gRNA 28L after 4 days of induction, representing an improvement over the original CRISPR-X (AID*Δ and M13 gRNA scaffold) construct performance in yeast of 26-fold (Figure 4C). When a gRNA that is less favorable to the Mtx2 scaffold, 29R, was used, the improvement was still 1.5-fold. To precisely quantify the mutational rate afforded by the yDBE platform, as well as to begin to understand this gRNA target site preference, we performed high-throughput amplicon sequencing of wtGFP pooled DNA mutagenized by the Mtx2 scaffold and the AID731Δ variant. More specifically, we targeted wtGFP with the AID731Δ-Mtx2 yDBE for 8 days to allow mutations to accumulate, then extracted genomic DNA, amplified the GFP locus, and sequenced it with an Illumina MiSeq instrument. To better generalize our conclusions, we used five separate GFP-targeting gRNAs at a variety of locations, with two targeting the template strand (t22L and t78R) and three targeting the coding strand (81L, 28L, and 29R). The gRNAs each performed at varying levels, with the use of t78R resulting in the fewest mutations and 29R resulting in the most (Supplemental Figure S4). We combined the results into a single plot using the 3′ end of the PAM as a reference point. Note that the data for the gRNAs that target the template strand were flipped due to the opposite directionality of the PAM. When combined, a clear mutation profile emerged, with substitutions appearing throughout a window that spanned approximately ±50 bp relative to the PAM (Figure 4D). In this window, the average rate of substitutions for the five Mtx2 gRNAs with the AID731Δ yDBE was 4.4 × 10^–3^ substitutions/bp. As approximately 20.5 yeast doublings (generations) occurred during the 8-day yDBE induction (Supplemental Figure S5), we estimate that the overall average rate of mutation is therefore 2.13 × 10^–4^ s.p.b. across a 100-nucleotide window centered slightly 5′ of the PAM and encompassing the gRNA targeting site. The rate of mutations at S65 for 28L and 29R correlated well with what we saw in the fluorescence shift assay (Figure 4C, Supplemental Figure S4). Nearly all of the substitutions occurred at CG pairs (Figure 4E), which is consistent with cytidine deaminase activity. The frequency of indels was extremely low, with the highest rates at any position reaching only 0.005% in the ±50-bp window. With the 29R gRNA, over 50% of reads had at least one mutation, and over 17% had two or more mutations (Supplemental Figure S4). These results confirm that our enhanced yDBE introduced a sufficient variety and magnitude of mutations to create screening libraries for directed evolution.

### Improving Antibody Affinity via yDBE *In Situ* DNA Diversification and Surface Display

We next demonstrated the capabilities of yDBE by using it to improve the affinity of an antibody. As a proof of concept, we integrated 4-4-20, a single-chain variable fragment (scFv) that binds fluorescein, into the genome of yDBE-expression yeast strains. Because they demonstrated differential activity in gRNA-spacer selection, we used both M13 and Mtx2 MS2 loop configurations, both in concert with the AID731Δ variant, in parallel directed evolution trials.

**Figure 5:**
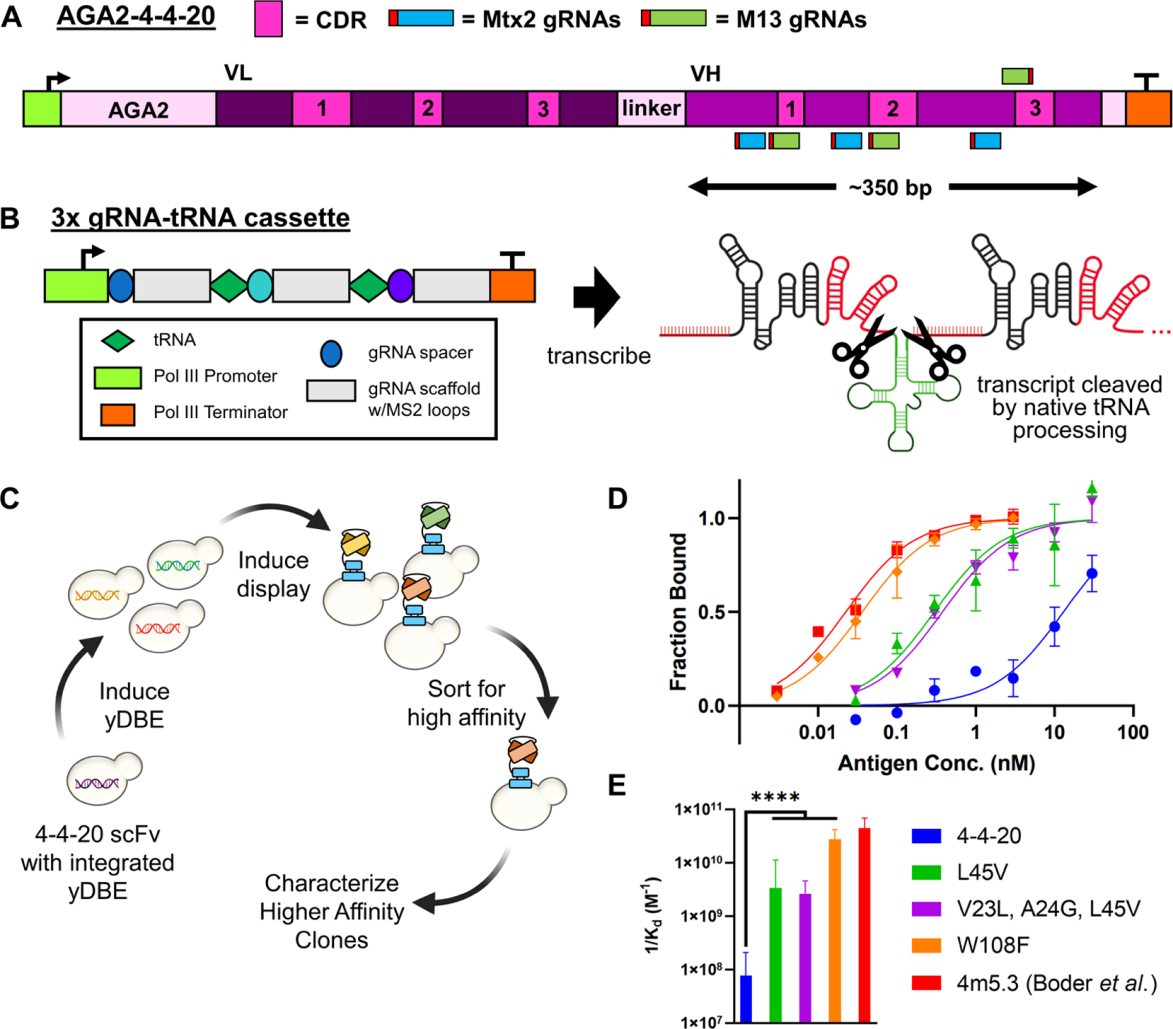
yDBE-mediated diversification and isolation of an improved scFv. (A) Map of AGA2-4-4-20 locus with CDRs and gRNAs highlighted. Two sets of gRNAs were created: one designed for M13 and the other for Mtx2. (B) Schematic of multiplexing three gRNAs by designing a gRNA-tRNA cassette. After transcription, the gRNAs were cleaved from the transcript as the cells processed the tRNAs with native RNases. (C) Process outline for improving antibody affinity using yDBE, yeast display, and FACS. (D) Antigen titration curves for wild-type 4-4-20, the three isolated mutants, and the ultrahigh affinity 4m5.3 variant. (E) Plot of inverse *K*_d_ for wild-type 4-4-20, the three isolated mutants, and the ultrahigh affinity 4m5.3 variant. *K*_d_ values were derived from the best-fit lines shown in D. A higher value indicated stronger binding. In E, bars represent a 95% confidence interval. ****p < 0.0001.

An scFv is composed of a V_H_ and V_L_ segment, each approximately 345 bp in length (Figure 5A). Therefore, it would not be possible to reach a high level of mutagenesis over the entire 4-4-20 DNA sequence using a single targeting gRNA with the yDBE, which can diversify across a region of ∼100 nucleotides. Complementarity determining regions (CDRs) within antibody heavy chains are particularly important for affinity, making it ideal if the *in situ* DNA diversification rate could be maximized within the three V_H_ CDRs of 4-4-20. To enable this function, we coexpressed three gRNAs as gRNA-tRNA arrays, allowing for multiple DNA sequence targeting.^49^ We created two separate, 3× gRNA-tRNA cassettes to target the V_H_ CDRs—one using M13 scaffolds and one Mtx2 (Figure 5B). Based on our results from the fluorescence shift assays and high-throughput sequencing, we targeted the coding strand ∼20 bp upstream (relative to the direction of transcription) of each CDR for the Mtx2 configuration. For the M13 configuration, we targeted the coding strand more directly on each CDR, except for CDR3 where we targeted the template strand due to the lack of a suitable PAM site on the coding strand.

We allowed 8 days in total for yDBE-mediated *in situ* antibody diversification to occur, with passages every 2 days (Figure 5C). After induction, we submitted amplicons from the yeast for high-throughput sequencing, which showed substitutions spread across the V_H_ of 4-4-20 for both M13 and Mtx2 gRNA cassettes, though the rate per gRNA appeared lower than what we would expect based on the 1× gRNA targeting of GFP (Supplemental Figure S6). The rate of substitutions was overall higher for the Mtx2 cassette, but since the cassettes use different spacers, this comparison should be approached with caution. It also should be noted that 4-4-20 has a substantially lower GC content compared to the mammalian codon-optimized GFP we used (46% vs 61%) which may explain the decrease in base editing capability. After scFv mutant library creation, cells were sorted four times using a competitive stain^6^ in which aminofluorescein is used to compete with fluorescein in the 4-4-20 scFv, leading to better antibody discrimination than equilibrium staining (Supplemental Figure S7). Following primary staining with biotinylated fluorescein and the competitive aminofluorescein stain, the cells were stained with streptavidin-PE and AlexaFluor647 anti-c-myc antibody. Therefore, antigen binding was indicated by the PE signal, whereas AlexaFluor647 showed the relative expression of scFv on the cell surface, allowing assessment of the antigen binding capability of scFv-positive cells.

After the final round of sorting, we plated cells and picked individual colonies to assess their affinity through yeast display. For the yDBEs using both the M13 and Mtx2 scaffolds, we found several mutant scFvs that had a substantial increase in affinity over wild-type 4-4-20. Sequencing of single scFvs showed mutations in each CDR of the heavy chain, near or overlapping the spacers we had selected (Table 1). The nucleotide substitutions all occurred at C or G residues, consistent with AID activity and our high-throughput sequencing results. Interestingly, certain mutants, such as L45V, were isolated from both the M13 and Mtx2 sorts, demonstrating convergence between the two libraries, despite having different gRNA spacer sequences. Three mutants were selected for further characterization: W108F isolated from the yeast using the M13 scaffold design, and L45V and V23L, A24G, L45V from the yeast harboring Mtx2 scaffolded yDBE, where residue numbering refers to the position within the V_H_. These three variants were amplified from genomic DNA and recloned into EBY100 to ensure that we were examining the scFv mutations in isolation. Using flow cytometry, we calculated each scFv’s *K*_d_ value for fluorescein by titrating a broad range of concentrations (Figure 5D). The W108F variant had a large 358-fold improvement over the 4-4-20 antibody (Figure 5E, Supplemental Table S2). By our screen, this is approaching the *K*_d_ of the high affinity, previously described 4m5.3 mutant.^6^ Interestingly, while the L45V variant has not been described previously, mutating the W108 residue has been shown to be productive for enhancing affinity, however the W108F mutation specifically did not stand out.^50^ In addition, affinity-enhancing mutations near multiple CDRs of the 4-4-20 scFv were isolated from a single library, suggesting the successful targeting of multiple DNA loci simultaneously using yDBE in single cells. As the L45V and V23L, A24G, L45V mutants also had improvement in affinity at 43-fold and 34-fold, respectively (Figure 5E), we demonstrated the ability to rapidly improve an antibody sequence using multiple yDBE designs by successfully isolating a variety of enhanced 4-4-20 variants.

**Table 1:** List of 4-4-20 Mutant Clones Collected after FACS

sorted clone	mutations	gRNA scaffold type	relative fluorescence	proximal CDR(s)	codon nucleotide changes
1^a^	W108F	M13	6.71	CDR3	TGG to TTT
2	L45V	M13	2.54	CDR2	CTG to GTG
3	L45V	M13	2.34	CDR2	CTG to GTG
4	W47L	M13	2.25	CDR2	TGG to TTA
5^a^	V23L, A24G, L45V	Mtx2	3.07	CDR1 and CDR2	GTT to CTT, GCC to GGC, and CTG to GTG
6	V23L, A24G, L45V	Mtx2	2.45	CDR1 and CDR2	GTT to CTT, GCC to GGC, and CTG to GTG
7	L45 (silent), W47L	Mtx2	1.99	CDR2	CTG to CTC, and TGG to TTA
8^a^	L45V	Mtx2	1.91	CDR2	CTG to GTG

a Clones taken for further analysis. Relative fluorescence values are mean antigen binding (PE) of scFv-expressing cells relative to wild-type 4-4-20.

## Conclusion

In this work, we designed and enhanced the mutational rate of yDBE, a CRISPR diversifying base editor for the *in situ* diversification of DNA in yeast. Using fluorescence shift-based assays, we improved and characterized two major components of the base editor. First, we universally improved yDBE mutagenesis rate 5-fold by surveying previously described and creating entirely new AID mutants with enhanced activity, particularly AID731Δ. Second, we assessed the mutational capability of a variety of gRNA/MS2 scaffold architectures and identified two, M13 and Mtx2, which support high rates of mutagenesis but have unique targeting preferences. In addition, by mutating either wtGFP or the 4-4-20 scFv using distinct gRNA regions, we demonstrated that yDBE can be reprogrammed to rapidly target new DNA sequences. Using high-throughput sequencing, we confirmed a variety of mutations occurring on both sides of the targeted spacer region, with the majority of mutations occurring within a 100-nucleotide window centered near the PAM. The combined Mtx2 mutation profile created through high-throughput sequencing showed a concentration of substitutions in the gRNA-binding region, especially at the 5′ end of where the spacer binds. dCas9 is relatively tolerant to single or sometimes even double mismatches in these areas, and for this reason we expect the base editor to continue operating despite the mounting mutations.^20,51^ We anticipate that the rate of base editing decreases at some point due to disruption of dCas9 binding, but in most cases, it could be much longer than the 4 or 8 day periods tested here.

We estimate that the enhanced yDBE employing the novel mutant AID731Δ in concert with the Mtx2 scaffold design has a mutation rate of 2.13 × 10^–4^ s.p.b. over a region of 100 nucleotides, which is comparable to previously described *in situ* mutagenesis platforms for yeast. Because of its ability to readily substitute C residues in both strands into any other nucleotide, the base editor can make a variety of mutations in many DNA sequences. There was a preference for C-to-G substitutions using yDBE which contrasts with results in CRISPR-X, carried out in mammalian cells, that showed a preference for C-to-T substitutions.^34^ This is likely due to the preference yeast have to insert a cytosine across from an abasic site during the translesion synthesis step of base excision repair.^52,53^ Indeed, Target-AID, a precise base editor employed in yeast, was similarly found to cause a high ratio of C-to-G substitutions in targeted poly-C regions, and polymerase η was identified as the most likely cause.^21,54^ Similarly, AID* (the triple mutant of wild-type AID) overexpression in yeast causes many C-to-G mutations, which required active base excision repair proteins UNG1 and REV1.^55^ In general, we saw a higher mutation rate when using gRNAs that targeted the coding strand. Targeting this strand with dCas9 alone has been shown to be mutagenic in yeast through R-loop formation in the transcribed strand, which exposes it to background deaminase activity.^41^ We hypothesize that this R-loop formation allows more access to the MCP-AID component of yDBE, leading to higher mutation rates.

One limitation of yDBE is the difficulty in universally mutating single, large genes >1,000 bp in length. While other systems such as OrthoRep and TRIDENT excel in this use case,^16,14^ they require placing genes adjacent to specific promoters, and these systems are unable to target multiple targets nor endogenous targets. Therefore, to further expand the targeting breadth of yDBE, we implemented a multiplexing gRNA expression cassette methodology by interspacing gRNAs with a tRNA. As the per-gRNA mutation rate for the 3× cassettes was lower than that for the 1× gRNAs targeting GFP, future work will focus on characterizing, confirming, and optimizing designs for multiplexing base editing. Interestingly, for both the M13 and Mtx2 gRNAs, the first gRNA of the cassette had the fewest substitutions near its target site, while the last gRNA had the most. This contradicts previous work with Cas9 gene knockout assays that found that the efficiency of the gRNAs generally decreased along the cassette.^49^ Targeting additional templates with new sets of spacers will elucidate any effect the array position has on gRNA efficiency. Another potential limitation of yDBE is the difficulty in targeting regions low in GC content. Our high-throughput sequencing determined that only 1.3% of mutations occurred at A or T bases when we applied our base editor. A way to overcome this limitation is to combine cytidine base editors with adenine base editors. Such a strategy has already been shown in CRISPR base editors in mammalian cells and plants^32,56^ and in a T7-RNAP-driven system (TRIDENT) in yeast.^14^ Another challenge with targeting areas low in GC content is the lack of the PAM sequences necessary to target the base editor. Utilizing a more promiscuous NG dCas9 would ameliorate issues of low PAM frequency.^28^

While we demonstrated the base editor by targeting exogenous genes (wtGFP and 4-4-20 scFv), it may be equally suitable for targeting endogenous genes. For this reason, we believe that our yDBE platform can be extended to a wide variety of directed evolution tasks. For instance, since the yDBE system appears to be amenable to multiplexing, it might aid in the evolution of more complex phenotypes such as resistance to stress and optimization of metabolic pathways by enabling the mutation of multiple distant loci simultaneously. Lastly, because our yDBE system uses AID, the mutagenic component driving somatic hypermutation in B cells, it might be engineered to better recapitulate the mutational profile of affinity matured antibodies compared to other *in vivo* mutagenesis systems. For this reason, we chose to demonstrate the ability of our system to improve the antibody affinity. In future work, yDBE might also be utilized to mutate naive antibody repertoires, more fully mimicking the process of affinity maturation.

## Methods

### Media, Culture, and Base Strains

NEB 10-beta *E. coli* (New England Biolabs) were used to amplify plasmid constructs for molecular cloning. *E. coli* were cultured in 5 mL of LB broth (Teknova) at 37 °C overnight with agitation. LB was supplemented with 34 μg/mL chloramphenicol (Sigma-Aldrich) or 100 μg/mL ampicillin (Sigma-Aldrich) antibiotic for selection.

All yeast strains developed in this work are derived from strain EBY100 (Leu^–^, Trp^–^; ATCC MYA-4941), which is designed for yeast display.^57^ When harboring a plasmid, yeast were cultured in 2 mL of synthetic glucose (dextrose) or galactose-Trp media (SD-Trp, or SG-Trp), comprised of 0.74 g/L complete supplemental media-TRP (CSM-TRP, Sunrise Science), 6.7 g/L yeast nitrogen base (YNB, BD), and 20 g/L glucose (Fisher Scientific) or galactose sugar (Sigma-Aldrich). In instances using selections for the Leu2 gene, CSM-TRP was replaced with 0.69 g/L CSM-LEU (Sunrise Science). When no selection was applied, YPD media, 10 g/L yeast extract (Thermofisher), 20 g/L peptone (Thermofisher), and 20 g/L glucose, was used. As needed, YPD was supplemented with 100 μg/mL of nourseothricin (Gold Biotechnology) for NAT gene selection. For yeast display of antibody fragments, SD-Trp or SG-Trp media was further buffered to pH 6.25 by adding 5.4 g/L Na_2_HPO_4_ and 8.56 g/L NaH_2_PO_4_·H_2_O.^58^ Yeast were grown at 30 °C with agitation. For both yeast and *E. coli*, solid media plates were made with the addition of 20 g/L of agar (Fisher Scientific).

### General Cloning Procedures

Polymerase chain reaction (PCR) was carried out using KOD Hot Start DNA polymerase (Sigma-Aldrich). Custom DNA oligomers were synthesized by Eurofins Genomics. All oligomers and primers are listed in Supplemental Table S3. Gibson Assembly was carried out using a master mix containing Taq Ligase (Enzymatics), Phusion polymerase (New England Biolabs), and T5 Exonuclease (New England Biolabs). 100 ng of linearized backbone was combined with a 2× molar excess of PCR inserts in a 5 μL volume. Fifteen μL of master mix was then added, and the reaction was run on a thermocycler at 50 °C for 1 h.

Golden Gate Assembly was carried out using a modification of previously described protocols.^49^ When annealing complementary oligos, compatible primers were combined at 25 μM in a 20-μL volume and held at 97 °C for 5 min, then ramped down to 20 °C over the course of 35 min. In a 20-μL reaction, 100 ng of base plasmid, 0.25 pmol of annealed oligos (or a 2x molar excess of insert when assembling gRNA-tRNA cassettes or HR plasmids), 2 μL of T4 Ligase 10× Buffer, 0.4 μL of T4 Ligase (New England Biolabs), and 1 μL of BsaI-HFv2 (New England Biolabs) were combined. The following temperature profile was used for the reaction: Step 1, 37 °C for 30 min; Step 2, 37 °C for 10 min; Step 3, 16 °C for 5 min; Step 4, repeat steps 2 and 3 for 30 cycles; Step 5, 37 °C for 30 min; Step 6, 60 °C for 5 min; Step 7, 80 °C for 5 min; Step 8, 4 °C hold. After assembly, the reaction mixture was dialyzed against ultrapure water and then transformed via electroporation into *E. coli* using standard electroporator protocols and then plated on solid media. Transformants were cultured overnight, and plasmids were extracted using a Qiaprep Spin Miniprep Kit (Qiagen). Plasmids were confirmed via both a restriction enzyme digest check and Sanger sequencing.

### Cloning Yeast Diversifying Base Editor (yDBE) Constructs

The amino acid sequence for MCP-AID*Δ^34^ was codon optimized for expression in yeast and synthesized by Twist Bioscience. MCP (MS2 phage coat protein) contains the N55K mutation^59^ and AID*Δ is an engineered version of human AID with the following amino acid mutations: K10E, T82I, E156G, 195*.^34^ MCP and AID*Δ are connected by a (GGGGS)^4^ linker and SV40 nuclear localization sequence. Our dCas9 construct was derived from plasmid bRA77 (Addgene plasmid # 100953) and includes a yeast-codon-optimized Cas9 from *Streptococcus pyogenes* with a triple, C-terminal, SV40 nuclear localization sequence.^60^ We used PCR and Gibson Assembly to introduce the necessary mutations (D10A and H840A) to make nuclease dead Cas9 (dCas9).

Both dCas9 and MCP-AID*Δ were first placed into base “EMY” constructs using Gibson Assembly. Promoters and terminators were then added to each sequence and cloned with Golden Gate Assembly into a backbone that is compatible with yeast homologous recombination (HR). Base EMY plasmids containing verified yeast promoters, terminators, and backbones (both HR-ready and 2μ expression plasmid sets) compatible with Golden Gate cloning were a gift from Eric Young.^61,62^ MCP-AID*Δ was placed under the control of the *S. cerevisiae* GAL2 promoter, while dCas9 was placed under the control of the GAL1 promoter. Both the GAL1 and GAL2 promoters are strongly induced in galactose media.

The 4-4-20 scFv fused to AGA2 was taken from plasmid pCT302 (Addgene plasmid # 41845) and placed in an HR vector.^63^ 4-4-20 is expressed under control of the pGAL1 promoter. Mammalian-codon-optimized wild-type GFP (wtGFP) was created from an eGFP expression vector, pcDNA3-eGFP-LIC (Addgene plasmid # 40768).^64^ Mutations L64F and T65S (reverting eGFP to wtGFP) were introduced using Gibson Assembly. Note that wtGFP still contains an H231L mutation and valine insertion at the “1a” position relative to *Aequorea victoria* GFP, but neither mutation affects the excitation/emission spectra.^40^ wtGFP was placed into a base EMY vector, and Golden Gate Assembly was used to place it in an HR vector along with a strong, constitutive promoter (pTDH3).

The sequence for AIDdead was synthesized as a linear fragment by Twist Bioscience, inserted into an EMY base vector using Gibson Assembly, and then placed into an HR vector using Golden Gate Assembly. Alternate codon optimizations of AID^34,42^ were synthesized by Twist Bioscience, amplified and cloned using a similar pipeline to AIDdead. Mutants AID731Δ, AIDmono, AID*mono, and AID731mono were created by amplifying fragments of AIDdead or AID*Δ with custom primers to introduce the desired mutations and then inserting the amplicons into an HR vector using Gibson Assembly. A complete list of mutations from the wildtype AID sequence can be found in Supplemental Table S1. For strains including RFA3, the RFA3 coding sequence was amplified from yeast genomic DNA and then fused to the C-terminus of AID*Δ or dCas9 and placed in an EMY backbone using Gibson Assembly followed by Golden Gate Assembly to place it into an HR backbone. A similar strategy was used to fuse AID731Δ to the C-terminus of dCas9 in an EMY backbone to create dCas9-AID731Δ. An alternate sequence for MCP,^43^ dubbed MCPz, was synthesized by Twist Bioscience and then fused to AID*Δ and directly cloned into an HR backbone using Gibson Assembly. All AID mutants were placed under the pGAL2 promoter to allow comparison to the original construct. dCas9-RFA3 and dCas9-AID731Δ were under the control of the pGAL1 promoter.

### gRNA Plasmid Cloning

For single targeting gRNA plasmids, a Golden-Gate-compatible base plasmid was first constructed by using Gibson Assembly. Four gRNA scaffolds were synthesized by Twist Bioscience: No MS2, M13, M4, and Mtx2. Each of these were cloned into a 2μ, Trp-selection plasmid, pY120,^62^ using Gibson Assembly creating pY120g-NoMS2, pY120g-M13, pY120g-M4, and pY120g-Mtx2, respectively. Each plasmid consists of a strong yeast RNA polymerase III pSNR52 promoter, a blank gap region flanked by BsaI cut sites, the gRNA scaffold variant, and a tSUP4 terminator. Using these first four plasmids, all the remaining gRNA scaffold variants were made using PCR and Gibson Assembly of partial scaffold fragments (M1, M14, M3tx2, etc.; Supplemental Table S4). To construct true targeting cassettes, e.g., to mutate wtGFP by targeting distinct DNA sequences within the gene as described below, the blank gap region was routinely replaced by a 20-bp spacer sequence using annealed oligos and Golden Gate Assembly. A full list of spacer sequences can be found in Supplemental Table S5.

For 3× gRNA-tRNA cassettes, the assembly strategy of GTR-CRISPR was generally followed.^49^ First a base plasmid was made to attach the M13 or Mtx2 scaffold gRNA with yeast tRNA^GLY^ (GCC). Gibson Assembly was used to join the tRNA to the C-terminal end of the gRNA scaffold in a pUC19 base vector, creating pUC19-M13-tRNAGly and pUC19-Mtx2-tRNAGly. At the C-terminal end of the gRNA scaffold, the tRNAs were separated by a short “AAACAA” nucleotide linker. We then used custom primers to perform two separate PCRs that would add the desired spacer sequences along with BsaI recognition sites that would reveal customized 4-bp gates when digested. Golden gates were checked for compatibility using a custom python script and a data set that measured gate fidelity in the presence of T4 Ligase.^65^ The two PCRs were combined with their matching pY120g-M13 or pY120g-Mtx2 base plasmids in a Golden Gate Assembly to produce a 3× gRNA-tRNA cassette.

### Yeast Strain Engineering

The Ura3 selection marker, along with the adjacent pGAL1-AGA1 construct, was removed by plating on 5-FOA to create strain EBY101 (Table 2), which was sequence confirmed following gDNA extraction and then used as a base for all fluorescence shift assay and wtGFP high-throughput sequencing tests. Linear fragments to be used for integration were amplified using PCR. For simultaneous integrations, each linear fragment had 50–60 base pairs of homology to the adjacent fragments (e.g., HR1 has homology to HR2 and HR2 has homology to HR3, etc.), as described previously.^62^ Linear fragments were integrated using the high-efficiency, lithium acetate transformation method,^66,67^ and integration loci were selected based off prior work suggesting sites that yield robust gene expression.^39^ For an initial strain construction step and demonstration of yDBE activity via mutation of wtGFP (shift assay described below), wtGFP was inserted at YORWΔ22 along with the NAT (nourseothricin resistance) gene (EBY101-wtGFP). The base editor gene expression constructs (e.g., MCP-AID*Δ and dCas9) along with a Leu marker gene were integrated simultaneously at YPRCτ3 (AC001). AIDdead was integrated in a similar manner (AC002). These integrations, along with all those described below, were confirmed by PCR of extracted genomic DNA. Finally, gRNA plasmids were transformed into desired strains using the same lithium acetate transformation protocol used for integrations.

**Table 2:** Description of Strains Constructed in This Work^a^

strain	base	modifications to base strain
EBY101	*EBY100*	Deletion of URA3 disruption cassette via collapse of flanking AGA1 sequences
EBY101-wtGFP	*EBY101*	YORWΔ22Δ::pTDH3-**mwtGFP**-tCYC1, psmTEF1-snNAT-tCYC1
AC001	*EBY101-wtGFP*	YPRCτ3Δ::pGAL2-MCP-AID*Δ-tVMA2, pagTEF1-klLEU2-tagTEF1, pGAL1-dCas9-tRPL3
AC002	*EBY101-wtGFP*	YPRCτ3Δ::pGAL2-MCP-**AIDdead**-tVMA2, pagTEF1-klLEU2-tagTEF1, pGAL1-dCas9-tRPL3
AC003	*EBY101-wtGFP*	YPRCτ3Δ::pGAL2-MCP-**AID731Δ**-tVMA2, pagTEF1-klLEU2-tagTEF1, pGAL1-dCas9-tRPL3
AC004	*EBY101-wtGFP*	YPRCτ3Δ::pGAL2-MCP-**AID731Δ**-tVMA2, pagTEF1-klLEU2-tagTEF1, pGAL1-**dCas9RFA3**-tRPL3
AC005	*EBY101-wtGFP*	YPRCτ3Δ::pGAL2-MCP-**dCas9AID731Δ**-tVMA2, pagTEF1-klLEU2-tagTEF1
AC201	*EBY101-wtGFP*	YPRCτ3Δ::PSNR52-**18L-M13**-TSUP4, psmTEF1-TRP1-tagTEF1, pGAL1-dCas9-tRPL3
AC202	*EBY101-wtGFP*	YPRCτ3Δ::PSNR52-**t22L-M13**-TSUP4, psmTEF1-TRP1-tagTEF1, pGAL1-dCas9-tRPL3
AC203	*EBY101-wtGFP*	YPRCτ3Δ::PSNR52-**NT1-M13**-TSUP4, psmTEF1-TRP1-tagTEF1, pGAL1-dCas9-tRPL3
EBY100-4420	*EBY100*	YORWΔ22Δ::pGAL1-**AGA2-4420**-tMF(ALPHA)1, psmTEF1-snNAT-tCYC1
AC301	*EBY100-4420*	YPRCτ3Δ::pGAL2-MCP-**AID731Δ**-tVMA2, pagTEF1-klLEU2-tagTEF1, pGAL1-dCas9-tRPL3
EBY100-4m5.3	*EBY100*	YORWΔ22Δ::pGAL1-**AGA2-4m5.3**-tMF(ALPHA)1, psmTEF1-snNAT-tCYC1
EBY100-W108F	*EBY100*	YORWΔ22: pGAL1-**AGA2-4420-W108F**-tMF(ALPHA)1, psmTEF1-snNAT-tCYC1
EBY100-L45V	*EBY100*	YORWΔ22Δ::pGAL1-**AGA2-4420-L45V**-tMF(ALPHA)1, psmTEF1-snNAT-tCYC1
EBY100-V23L, A24G, L45V	*EBY100*	YORWΔ22Δ::pGAL1-**AGA2-4420-V23L,A24G,L45V**-tMF(ALPHA)1, psmTEF1-snNAT-tCYC1

a Abbreviations: ag = *Ashbya gossypii*, kl = *Kluyveromyces lactis*, sm = *Saccharomyces mikatae*, sn = *Streptomyces noursei*. All other promoters/terminators are from *Saccharomyces cerevisiae*.

To test MCP-AID mutants, three preliminary strains were created that had an integrated wtGFP at YORWΔ22 and an integrated dCas9 and gRNA expression cassette (either 18L, t22L, or NT1 gRNA targeting sequence) at YPRCτ3 (strains AC201–203). Then, for each MCP-AID variant, linear expression constructs were amplified and integrated at the YPRCΔ15 locus^39^ with TRP selection in strains AC201–203. For the sake of brevity, the resultant strains are excluded from Table 2. For further analysis, MCP-AID731Δ and dCas9 were later integrated at YPRCτ3 (AC003), which facilitated comparisons to AC001 with a larger set of gRNAs expressed on plasmids. dCas9-RFA3 with AID731Δ or dCas9-AID731Δ*w*ere integrated at YPRCτ3 (AC004 and AC005, respectively), followed by gRNA plasmid transformation, for comparison with AC003.

For creation of strains for yeast display studies and mutation of an scFv, an AGA2-4-4-20 expression construct was inserted at YORWΔ22 along with the NAT gene in EBY100. The optimized base editor (MCP-AID731Δ and dCas9) was then integrated into YPRCτ3 (AC301). Lastly, the M13 and Mtx2 3× gRNA-tRNA plasmids were transformed into this strain. To confirm the binding profile of 4-4-20 variants, 4-4-20 mutant strains were created by first making an integration-compatible vector using Gibson Assembly, then amplifying a linear AGA2-4-4-20 (mutant) fragment using PCR and integrating the construct into the otherwise unmodified strain EBY100.

### wtGFP-eGFP Fluorescence Shift Assay

For the wtGFP-eGFP fluorescence shift assay, yeast were picked from a plate into 2 mL of SD-Trp at 30 °C with shaking. After overnight growth, the cells were induced by diluting the cells down to an OD of 0.25 in 2 mL of SG-Trp media. Cells were then cultured for a specified time (1–8 days). For inductions longer than 2 days, cultures were passaged in fresh SG-Trp media every 2 days, with the initial OD set at 0.25. After induction of yDBEs in galactose, 1 × 10^7^ cells were rinsed with phosphate buffered saline (PBS) and analyzed using a FACSMelody flow cytometer (BD). Flow cytometry data analysis was performed using FlowJo.

### High-Throughput Sequencing of Mutated Genomic Yeast DNA

To induce mutations prior to high throughput sequencing, AC003 yeast with pY120g-Mtx2–28L was cultured for 8 days in SG-Trp media. The cells were diluted every 2 days in fresh media to an OD of 0.25. Genomic DNA was collected by using the Yeastar Genomic DNA Kit (Zymo Research). The wtGFP locus was amplified by PCR with primers that added sequencing adapters, and DNA concentrations were measured by using the Qubit fluorimetry system. DNA was sent to Genewiz for EZ-Amplicon sequencing (PE250 MiSeq, Illumina), generating 100,000+ reads per run. The paired reads were first merged together using BBMerge and then aligned to the reference using bwa mem.^68,69^ Then, variant calls were compiled together using samtools mpileup.^70^ To remove the background signal from our analysis, DNA from EBY101-wtGFP with plasmid pY120 (which lacks all base editor components) was also sequenced. The cells were similarly cultured for 8 days, and the amplified DNA was prepared as similar to the base editor strains. Before the final substitution frequency analysis, the background signal from EBY101-wtGFP was subtracted from the signal collected from the base editor strain and negative values were set to zero. DNA from the V_H_ of 4-4-20 was prepared and sequenced similarly to GFP DNA.

A custom Python script was used to calculate and visualize the average substitution and insertion/deletion rate over a user-specified window. First, the substitution rate was calculated on a per-nucleotide basis using the file generated by mpileup. The rate is the number of reads with a mismatch from the reference nucleotide divided by the number of total reads that aligned to that nucleotide excluding insertions or deletions. These per-nucleotide rates could then be averaged across a window to give an overall substitution rate. Additional custom Python scripts were used to plot the frequency of mutations at each base (distribution plots) and to map the frequency of each type of substitution (heatmaps). The number of substitutions per read was calculated and visualized using a custom Python script that processed the MD:Z tag from the bam file produced during the bwa mem alignment step. All Python scripts are available upon request.

### Yeast Display and Sorting

To induce mutations prior to staining and sorting, yeast were cultured for 8 days in SG-Trp. The cells were diluted every 2 days in fresh media down to an OD of 0.25. Cells were induced to display by first growing in buffered SD-Trp media overnight then diluting the cells to OD 0.5 in buffered SG-Trp and culturing for 24 h.

Yeast display was performed following established protocols.^58^ Due to the relatively high starting affinity of 4-4-20 for fluorescein, the scFvs were screened using a competitive assay.^6^ 2 × 10^7^ cells were first rinsed with PBSF (PBS with 0.1% BSA) and stained with 1 μM biotinylated fluorescein (Biotium) for 60 min in a volume of 200 μL. Cells were rinsed again with PBSF then stained with aminofluorescein (Thermo Scientific), a nonfluorescent competitor, as described previously.^71^ Cells were then placed on ice and then rinsed with ice-cold PBSF and scFv expression was stained for using an anti-c-myc antibody conjugated to AF647 (Cell Signaling Technologies) at 8 μg/mL. The presence of remaining scFv-bound biotinylated fluorescein was visualized using streptavidin-PE (Invitrogen) at 10 μg/mL. The secondary stain was performed on ice in the dark for 30 min. The cells were then rinsed and sorted by using a FACSMelody instrument. The sorted cells were collected in SD-Trp media and allowed to recover for 1–2 days. This process was repeated four times, each time using a more stringent gate during FACS. After the fourth sort, the cells were plated on synthetic TRP plates and allowed to grow for 2 days.

Single yeast colonies were picked and compared against strain EBY100–4420. Clones that had a substantial increase in antigen binding in a competitive stain relative to EBY100–4420 were selected for further characterization. From these mutants, the 4-4-20 scFv gene was extracted using a nested colony PCR and Sanger sequenced. To verify that affinity improvements were definitively and solely from mutations in the 4-4-20 sequence, the mutant sequences were copied using PCR, cloned into an HR backbone, and integrated into an unmodified strain background, as described above.

### Titration of Antibody Affinity

An antigen titration was used to measure the affinity of 4-4-20 and its variants.^50^ Cells were first cultured and induced to display scFv as described above. 1 × 10^5^ cells were rinsed then stained in 500 μL of PBSF with antigen concentrations ranging from 0.3 pM to 30 nM of biotinylated fluorescein for 3 h at room temperature. For antigen concentrations 0.1 nM and 0.03 nM, 1 × 10^4^ displaying cells were mixed with 1 × 10^5^ of nondisplaying cells to both ensure that antigen quantities were never limiting and there were still sufficient cells to form a pellet. For the lowest two concentrations, 3 pM and 0.01 nM, 1 × 10^5^ cells were used, but the volume was increased to 40 and 14 mL respectively to prevent limiting antigen quantities. After primary staining, cells were placed on ice, rinsed with ice-cold PBSF, and then stained with secondary reagents (streptavidin-PE and anti-c-myc-AF647) in 30 μL for 30 min on ice. Cells were then rinsed and analyzed on a FACSMelody flow cytometer. Data was normalized and best-fit lines were calculated using a nonlinear regression in Graphpad Prism according to established protocols.^58^

### Statistics

All the fluorescence shift assays and the antigen titration were performed in biological triplicate (*n* = 3). All reported error bars represent one standard deviation, except where otherwise noted. To calculate *p*-values, a one-way ANOVA was performed followed by a multiple comparison test using Tukey’s range method in Graphpad Prism. For comparisons of the dissociation constants generated by the best-fit curves of the antigen titrations, an extra-sum-of-squares F test was done in Graphpad Prism.

## Supporting Information Available

Fluorescence shift assay description, FACS plots of fluorescence shift assays, additional fluorescence shift assay data, growth rate data, high-throughput sequencing data, FACS plots from yeast display sorting, AID mutant table, scFv *K*_d_ table, primer sequences, gRNA scaffold table, gRNA spacer table, and descriptions (PDF)
