# Supplementary material for: A Rapid Antibody Enhancement Platform in Saccharomyces cerevisiae Using an Improved, Diversifying CRISPR Base Editor
Source: ACS Synth Biol. 2023 Oct 24;12(11):3287–300. doi: 10.1021/acssynbio.3c00299 (PMC10661033; doi:10.1021/acssynbio.3c00299)
Supplement: Supplementary file 1 — sb3c00299_si_001.pdf [file sb3c00299_si_001.pdf]

Supporting Information for:

A rapid antibody enhancement platform in *Saccharomyces cerevisiae* using an improved, diversifying CRISPR base editor

Andrew P. Cazier[1], Olivia M. Irvin[1], Lizmarie S. Chávez[1], Saachi Dalvi[1], Hannah Abraham[1], Nevinka Wickramanayake[1], Sreenivas Yellayi[1], and John Blazeck[1]*

Author Contact Information:

[1] School of Chemical and Biomolecular Engineering, Georgia Institute of Technology, Atlanta, GA 30332, USA.

* Corresponding author: john.blazeck@chbe.gatech.edu

This file contains seven supplemental figures and five supplemental tables.

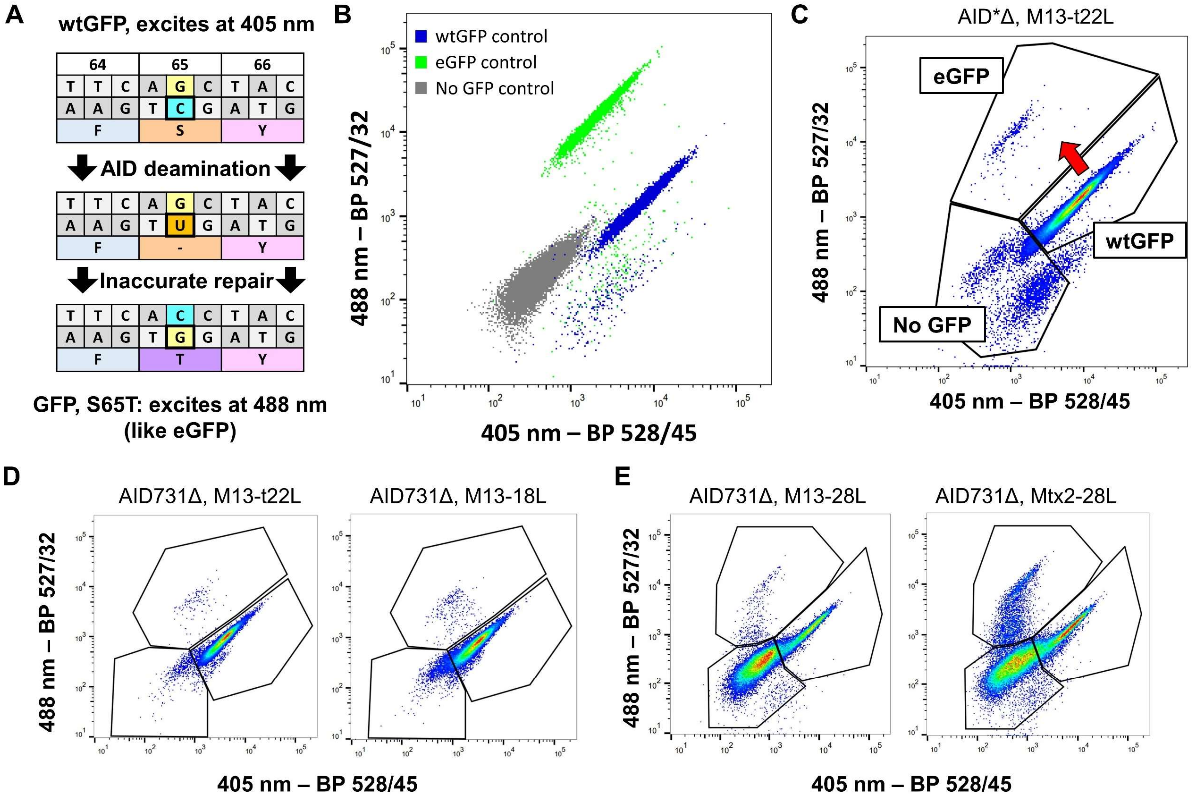

Supplemental Figure S1. Description of fluorescence shift assay and representative FACS plots. A) wtGFP contains an AGCT motif which is preferred by AID. Deamination and inaccurate repair can lead to the S65T mutation, which causes a shift in the excitation spectrum. **B)** Control populations showing how wtGFP, eGFP, and No GFP have essentially no fluorescence overlap. **C)** FACS plot showing that a population of eGFP-positive cells is generated after 8 days of base editing using strain AC001 with t22L gRNA plasmid. Compare with **Figure 1C**. **D)** FACS plot showing strain AC003 after 4-day induction with two, separate gRNAs, M13-t22L or M13-18L. Compare with **Figure 2**. **E)** FACS plot showing strain AC003 after 4-day induction with two, separate gRNAs, M13-28L or Mtx2-28L. Compare with **Figure 4C**. BP, band pass filter.

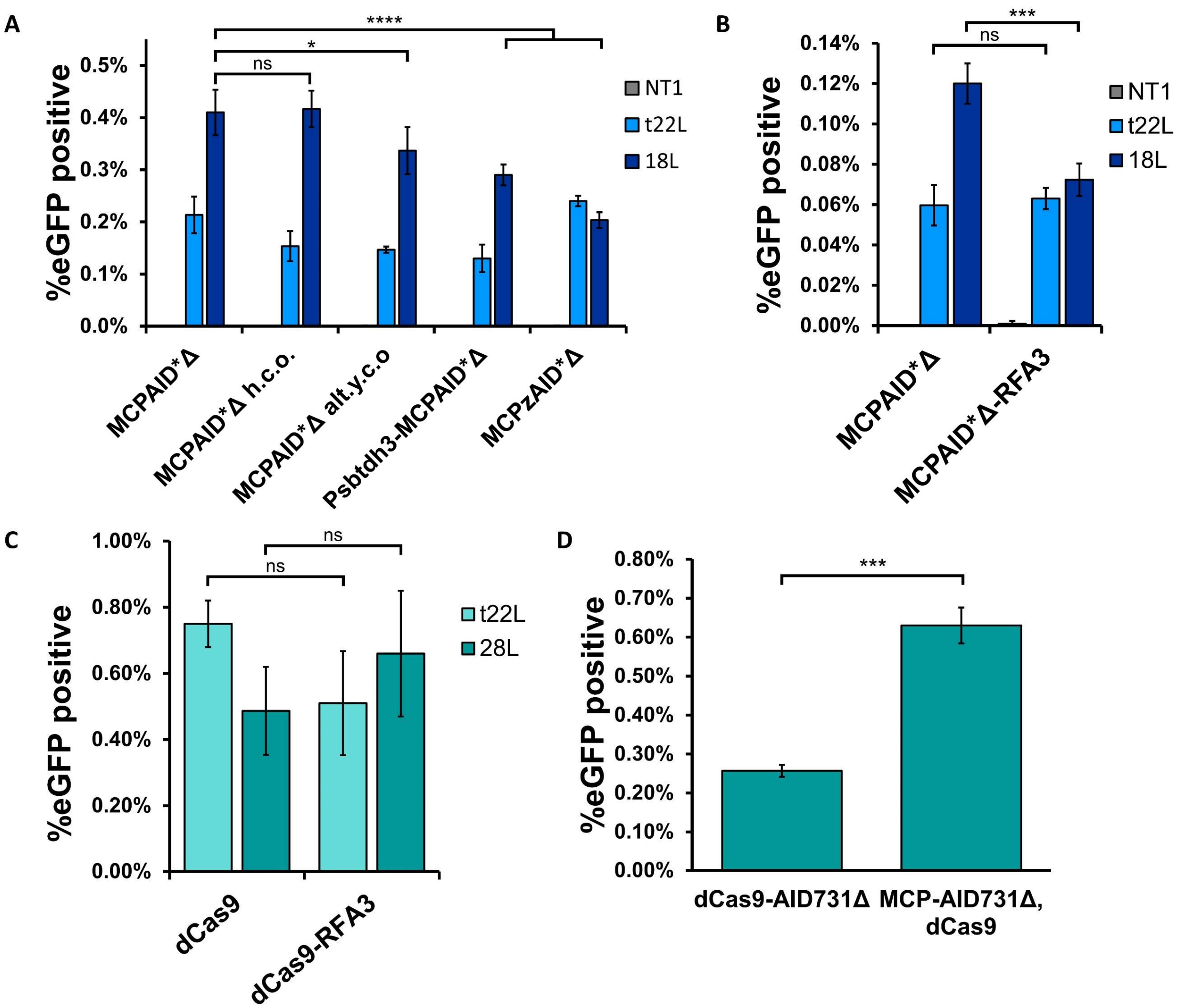

Supplemental Figure S2. Fluorescence shift assay results after modifying yDBE components. A) Fluorescence shift assay with variants or alterations of MCP-AID*Δ after a 4-day induction. h.c.o = human codon optimized, alt.y.c.o = alternative yeast codon optimization. MCPz is a variant of MCP. Psbtdh3 is a strong, constitutive promoter from *Saccharomyces boulardii*. All other variants are controlled by the *S. cerevisiae* GAL2 promoter. NT1 is a nontargeting gRNA; t22L and 18L are targeting. **B)** Fluorescence shift assay after 4-day induction with AID*Δ vs AID*Δ-RFA3. **C)** Fluorescence shift assay after 4-day induction with AID731Δ and dCas9 (strain AC003) or dCas9-RFA3 (strain AC004). M13 scaffolds were used for all gRNAs. **D)** Fusing AID directly to dCas9 (strain AC005), vs recruiting AID with MCP (strain AC003), did not improve the rate of mutation in a fluorescence shift assay when using an M13-28L gRNA. Cells were induced for 6d. In **A-D**, bars represent mean±SD, *n*=3, ns=not significant, *p<0.05, ***p<0.001, ****p<0.0001.

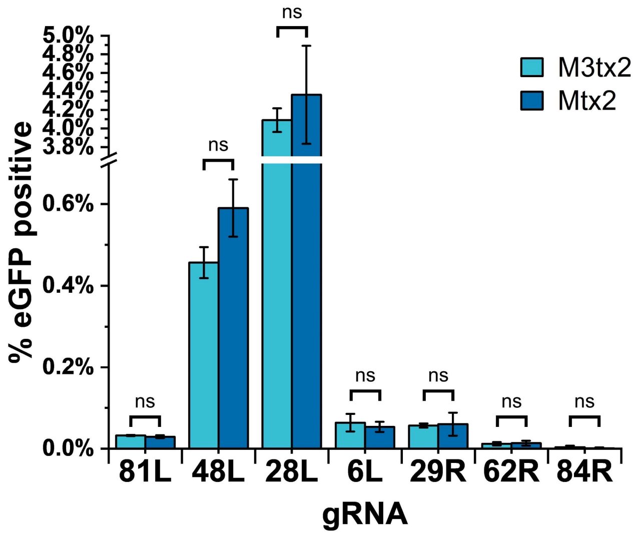

Supplemental Figure S3. Fluorescent shift assay gRNA tiling experiment comparing Mtx2 and M3tx2. Fluorescence shift assay after 4-day induction comparing M3tx2 and Mtx2 scaffolds with 7 positional spacers with AID*Δ (Strain AC001). Bars represent mean±SD, *n*=3, ns=not significant.

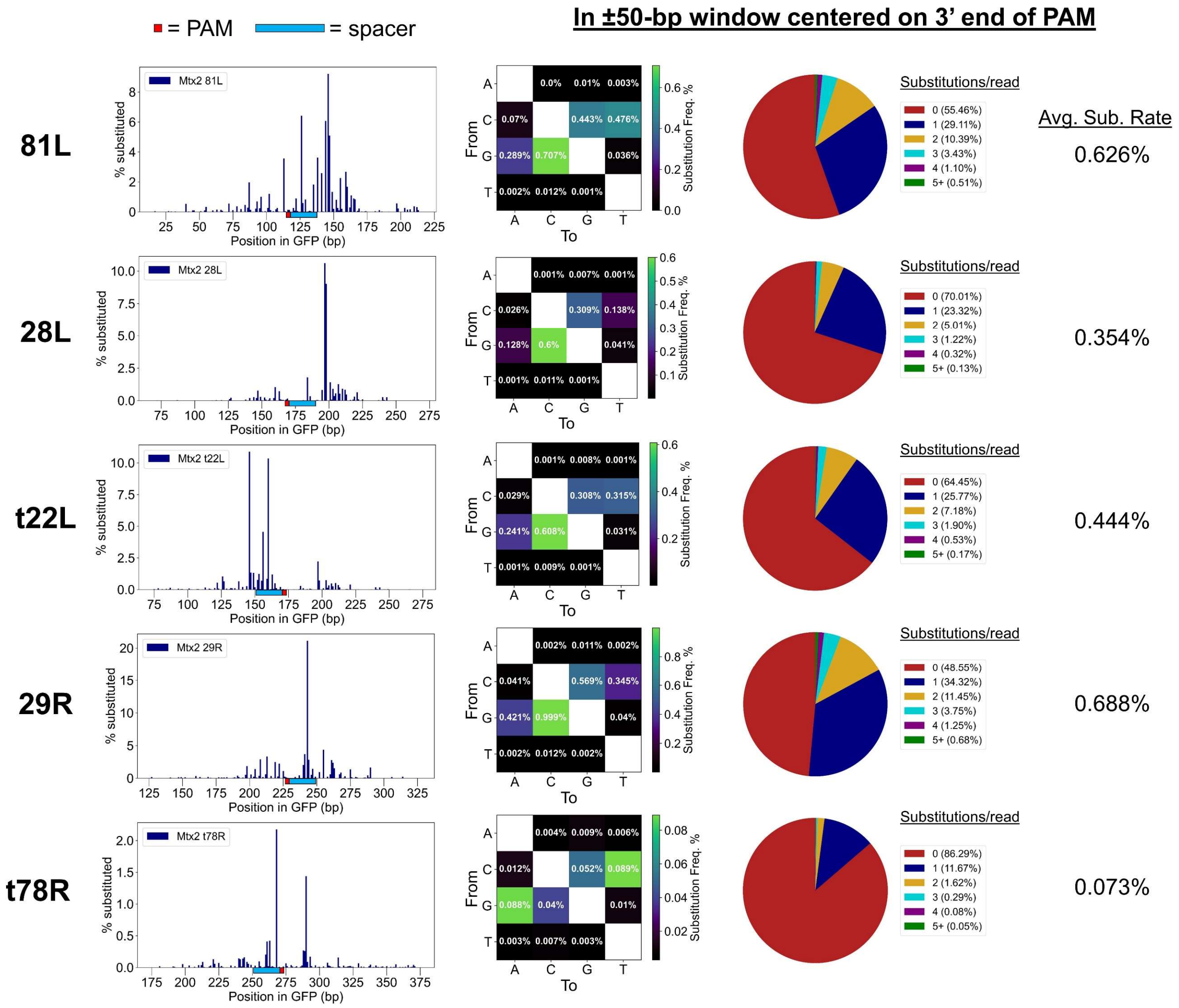

Supplemental Figure S4. High-throughput sequencing results for five separate, GFP-targeting Mtx2 gRNAs. DNA was extracted, amplified, and sequenced after 8-day yDBE induction in strain AC003. For each gRNA, a substitution distribution plot, substitution heatmap, and substitution-per-read pie chart were generated. The substitution distribution plot shows the per-nucleotide rate of substitutions in a ±100-bp window. The relative position and orientation of the gRNA is shown along the x-axis. The substitution heatmap shows the average rate at which each possible substitution type was detected in a ±50-bp window. The pie chart shows how many substitutions were detected in a ±50-bp window per sequencing read. Lastly, the average mutation rate in a ±50-bp window is given on the right.

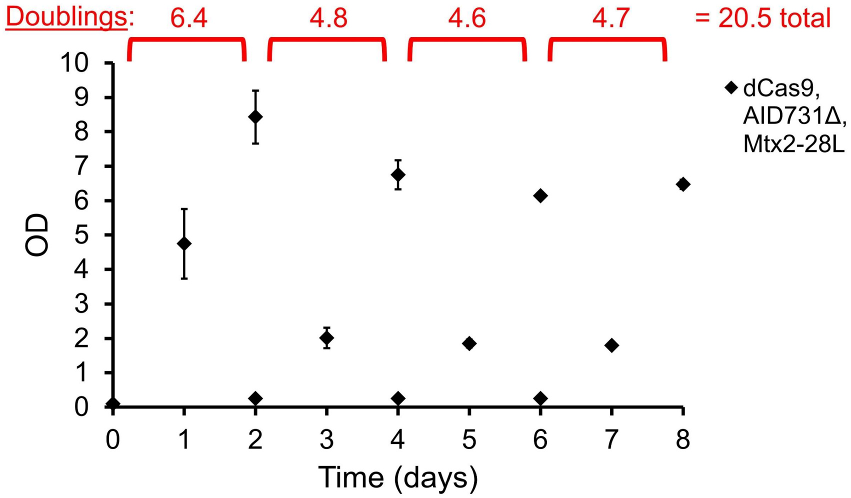

Supplemental Figure S5. Growth rate of yDBE yeast. Representative optical density (OD) measurements during 8-day galactose induction of strain AC003 with Mtx2-28L plasmid. At time zero, the cells began at an OD of 0.1. Every two days, they were passaged with fresh SD-Trp media and diluted to an OD of 0.2. Bars represent mean±SD, *n*=3.

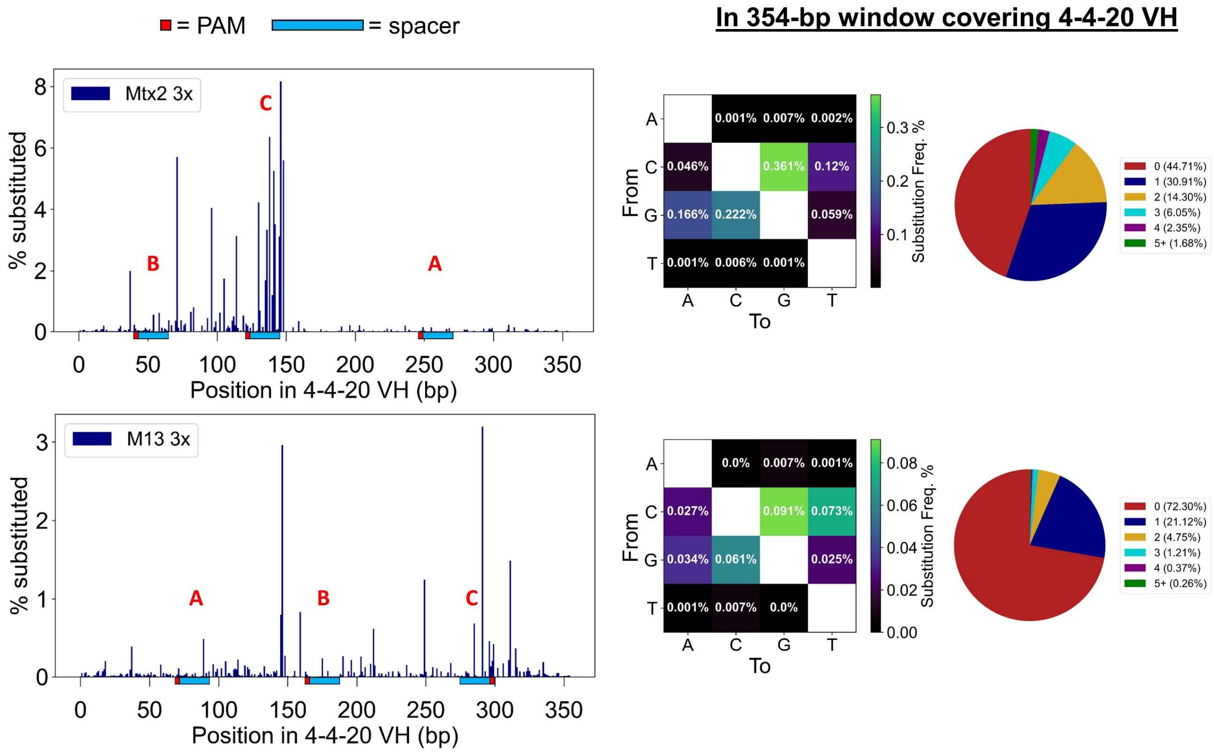

Supplemental Figure S6. High-throughput amplicon sequencing results of 4-4-20 V_H. DNA was extracted and amplified after 8-day yDBE induction in strain AC301 with a 3x gRNA-tRNA cassette using Mtx2 (top) or M13 (bottom) scaffolds. The substitution distribution plots show the per-nucleotide rate of substitutions in a 354-bp window covering the entire V_H of 4-4-20. The approximate position and orientation of each gRNA is shown along the x-axis, and the position of each gRNA within the 3x cassette (A, B, then C) is shown on the plot. The substitution heatmap shows the average rate a which each possible substitution type was detected within the 354-bp window. The pie chart shows how many substitutions were detected in the 354-bp window per sequencing read.

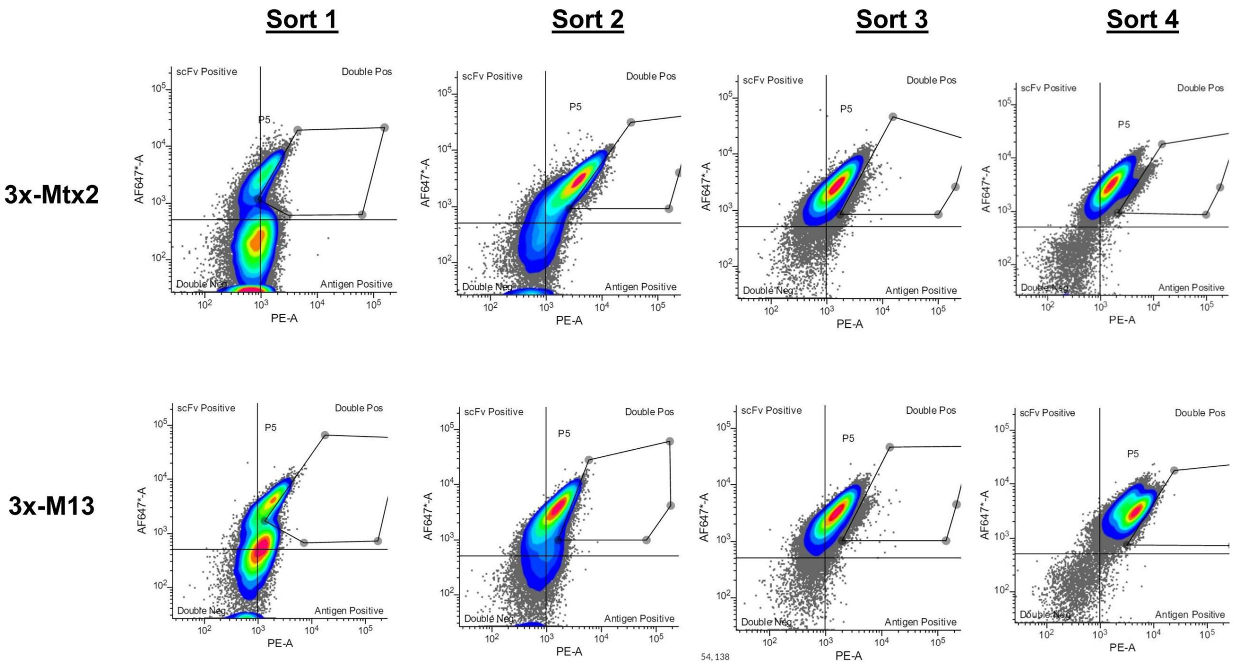

Supplemental Figure S7. FACS plots of 4-4-20 mutant sorting. FACS plots showing yeast display of 4-4-20 prior to each of four sequential sorts to select for high-affinity antibodies. Anti-c-myc with AF647 measures scFv expression (y-axis) while the antigen binding is measured with streptavidin-PE (x-axis) following a competitive stain to discriminate the best binders. Prior to Sort 1, AC301 yeast were induced to base edit for 8 days with a 3x gRNA-tRNA cassette with M13 or Mtx2 scaffolds.

Supplemental Table S1. AID mutants

Name	Mutations (wild type AID has 198 AA)
AID*Δ	K10E, T82I, E156G, 195*
AIDmono	(3S-K10) to PAT, L12T, H130A, R131E, 181*
AID*mono	(3S-K10) to PAT, L12T, T82I, H130A, R131E, E156G, 181*
AID731mono	(3S-K10) to PAT, L12T, R36C, L44R, T82I, Y88S, H93L, H130A, R131E, K142E, E156G, 181*
AID731Δ	R9S, K10E, R36C, L44R, T82I, Y88S, H93L, K142E, E156G, 195*
AIDdead	H56R, E58Q, 195*

Supplemental Table S2. K_d values based on antigen titration

scFv name	Calculated K_d (nM)	95% confidence interval (nM)
4-4-20	13.0	4.79 to 48.3
L45V	0.299	0.0891 to 1.02
V23L, A24G, L45V	0.379	0.220 to 0.667
W108F	0.0364	0.0240 to 0.0548
4m5.3	0.0225	0.0146 to 0.0340

Supplemental Table S3. Primers used in this study

NOTE: Primers are shaded in pairs used for PCR, except for the one-sided, nested PCRs, which have three primers. Repeated primers are highlighted in RED.

Primer#	Sequence (5' to 3')	PCR Purpose
	**Genomic integration primers**	
apc001	CGGTATTACTCGAGCCCGTAATAC	5' Flank for Integration @YPRCτ3
apc002	GGACACCTGGCTACTTAACCATTCGTTGTTAGTGTGTCGCATACGAGGAATAACGCCGATGGGACGTCAGCACTGTAC	
apc003	AAAGGAGGTGCACGCATTATGG	3' Flank for Integrating 3 Genes @YPRCτ3
apc004	CCGAACCTAGGATTAGATGTGGTCTAGCACCATATTGCGGACATGGTCCCCCTGTTATTCCAAGGAGGTGAAGAACGTC	
apc005	GGACACCTGGCTACTTAACCATTCGTTGTTAGTGTGTCGCATACGAGGAATAACGCCTTTGCGAAACCCTATGCTCTG	5' Flank for Integration @YPRCΔ15
apc006	GCCAGGCGCCTTTATATCATATAATTAAGAC	
apc007	CATTTGGATTGTAATTTCATACTGGAGTAAACATCTCCAGGTGTCTAAGTTCACACAGGAATGGAAGGTCGGGATGAGC	3' Flank for Integrating 2 Genes @YPRCΔ15
apc008	ATAAAGCAGCCGCTACCAAACAG	
apc009	CGTGATAAACGATCGCCATAACTAAC	3' Flank for Integration @YORWΔ22
apc010	GATTGTAATTTCATACTGGAGTAAACATCTCCAGGTGTCTAAGTTCACACAGGGGACCAACTATCATCCGCTAATTAC	
apc011	CACCGGAGCTTGGATATGATAAAC	5' Flank for Integrating 2 Genes @YORWΔ22
apc012	GGCTACTTAACCATTCGTTGTTAGTGTGTCGCATACGAGGAATAACGCCTTCGCGGGCTGTTACTTATCC	
apc013	GGCGTTATTCCTCGTATGCG	Amplify Pro-gene-Term in HR1 backbone
apc014	GACAATCGCTACAGAAACGATTTTC	
apc015	AGGACCAAGCGACCTGTGTC	Amplify Pro-gene-Term in HR2 backbone

apc016	CCTGTGTGAACTTAGACACCTGGAG	
apc017	TCATTTGGATTGTAATTTCATACTGGAG	Amplify Pro-gene-Term in HR3 backbone
apc018	TAACAGGGGGACCATGTCC	
apc019	ACGAGCTTTTGAATTATGGTAATTTTG	PCR check for integrations @YPRCΔ15
apc020	TGTTGAGTACTTCAACTTTATTTCCTTC	
apc021	CCGTGAATCAAGCTGATAAACAG	PCR check for integrations @YPRCτ3
apc022	CCTGGACACTTTACTTATCTAGCG	
apc023	GGAAATATATGCGCAGTATGCTCC	PCR check for integrations @YORWΔ22
apc024	CGAATCAAACGAATGCTTTGGAAAC	
apc025	GTTATTCCTCGTATGCGACACACTAACAACGAATGGTTAAGTAGCCAGGTGTCCATCCCAGTGAGTTGATTGGAAGACC	PCR pSNR52-gRNA-tSUP4, mimicks amplification from HR1 backbone
apc026	CAATCGCTACAGAAACGATTTTCAACAGTATTTACCTCGACACAGGTCGCTTGGTCCTGTGAGCTGATACCGCTCGAAG	

4-4-20 scFv and GFP

apc027	GTTATTCCTCGTATGCGACACACTAACAACGAATGGTTAAGTAGCCAGGTGTCCATCACATGGCATTACCACCATATAC	AGA2-4-4-20 for insertion
apc028	CTACAGAAACGATTTTCAACAGTATTTACCTCGACACAGGTCGCTTGGTCCTAATTCTCTTAGGATTCGATTCACATTC	
apc029	TACTTCTTATTCAAATGTAATAAAAGATCGAATTCCCTACTTCATACATTTTCAATTAAG	AGA2-4m5.3 Fragment 1 for pCT backbone
apc030	CTAGCAGAACCACCACCACCAGAAC	
apc031	GGTGGTGGTGGTTCTGCTAGCGACGTCGTTATGAC	AGA2-4m5.3 Fragment 2 for pCT backbone
apc032	GTTACATCTACACTGTTGTTATCAGATCTCGAGCTATTACAAGTCTTCTTCAGAAATAAGCTTTTG	
apc033	CAGAGCAGATTGTACTGGGTCTCAAATGGTGAGCAAGGGCGAGG	wtGFP Fragment 1 for EMY backbone
apc034	CGCCGTAGCTGAAGGTGGTCACGAGGGTGG	
apc035	GTGACCACCTTCAGCTACGGCGTGCAGTGCTTC	wtGFP Fragment 2 for EMY backbone
apc036	GAGCTGATACCGCTCGGTCTCTTTTACTTGTACAGCTCGTCCATG	
apc037	CATCAGAGCAGATTGTACTGGGTCTCAAATGCAGTTACTTCGCTGTTTTTC	AGA2-4-4-20 for EMY backbone
apc038	GTGAGCTGATACCGCTCGGTCTCTTTTACAAGTCTTCTTCAGAAATAAGCTTTTG	
apc039	AAAGGTCTCAGTGCACATGGCATTACCACCATATACATATCC	AGA2-4-4-20 mutants for HR backbone
apc040	AAAGGTCTCAGAGGAATTCTCTTAGGATTCGATTCACATTCATC	

High-throughput sequencing

apc041	ACACTCTTTCCCTACACGACGCTCTTCCGATCTATCACGAATGGTGAGCAAGGGCGAGGAGC	High-throughput sequencing of GFP
apc042	GACTGGAGTTCAGACGTGTGCTCTTCCGATCTCGATGTTCAGCTCGATGCGGTTCACCAGG	
apc151	ACACTCTTTCCCTACACGACGCTCTTCCGATCTGCATCAGATGGTGACGTCAAACTGGATGAGAC	High-throughput sequencing of 4-4-20
apc152	GACTGGAGTTCAGACGTGTGCTCTTCCGATCTTGAGACCATCTACACTGTTGTTATCAGATCTCGAGC	

dCas9 and fusions

apc043	CATCAGAGCAGATTGTACTGGGTCTCAAATGGATAAAAAGTATAGTATTGGTTTAGCTATTG	dCas9 Fragment 1 for EMY backbone
apc044	CAAGAAAGATTGTGGAACTATGGCATCTACATCATAATCTGAAAG	

apc045	CTTTCAGATTATGATGTAGATGCCATAGTTCCACAATCTTTCTTG	dCas9 Fragment 2 for EMY backbone
apc046	GAGCTGATACCGCTCGGTCTCTTTTAAACCTTTCTCTTTTTCTTAGGATCCAC	
apc047	GAGCTGATACCGCTCGGTCTCTTTTAGTATATTTCTGGGTATTTCTTACATAGTCTC	Nested PCR to fust RFA3 to dCas9 for EMY backbone, reverse primer
apc048	CTAGCGGATCCGAGACTCCTGGGACCTCAGAGTCTGCTACACCCGAAAGTTCAGGTGGATCTTCTGGTG	forward primer 1
apc049	GGTGGATCCTAAGAAAAAGAGAAAGGTTTCCGGTGGATCTTCTGGTGGTTCTAGCGGATCCGAGACTCC	forward primer 2
apc050	GAGCTGATACCGCTCGGTCTCTTTTATGTCCTGAATGCATCACGTAAATC	Nested PCR to fuse AID731Δ to dCas9 for EMY backbone, reverse primer
apc051	TTCAGGTGGAGGCAGTGGAGGTGGTGGATCTATGGATTCACTATTAATGAATAGAAGTG	forward primer 1
apc052	CGTAAGGTGGATCCTAAGAAAAAGAGAAAGGTTTCAGGTGGAGGCAGTGGAG	forward primer 2

AID variants (AID*Δ, AIDdead, AIDmono, AID*mono, AID731Δ, etc.)

apc053	CATCAGAGCAGATTGTACTGGGTCTCAAATGGCTAGTAATTTTACTCAATTCGTG	MCP-AID*Δ for EMY backbone
apc054	GAGCTGATACCGCTCGGTCTCTTTTATGTCCTGAATGCATCACGTAAATC	
apc053		MCP-AIDdead Fragment 1 (MCP) for EMY backbone
apc055	CTCTTTTTCTTAGGGCCTGAACC	
apc056	AGGCCCTAAGAAAAAGAGAAAAGTGG	MCP-AIDdead Fragment 2 (AIDdead) for EMY backbone
apc054		
apc053		MCP-AIDmono Fragment 1 (MCP) for EMY backbone
apc057	ATCCATGCCGGCTGCGGCCACTTTTC	
apc058	GAAAAGTGGCCGCAGCCGGCATGGATCCAGCTACCTTTACGTACCAATTTAAGAACGTGAGATGG	MCP-AIDmono Fragment 2 for EMY backbone
apc059	CATGGTGACCAAGAGGTAAACCATGTAACACGATAACAC	
apc060	GTGTTACATGGTTTACCTCTTGGTCACCATGCTATG	MCP-AIDmono Fragment 3 for EMY backbone
apc061	CTGAACTCCGGCTTCGGCTAATCTTCTTAAGCCTTCAGGC	
apc062	AGGCTTAAGAAGATTAGCCGAAGCCGGAGTTCAGATTGC	MCP-AIDmono Fragment 4 for EMY backbone
apc063	GAGCTGATACCGCTCGGTCTCTTTTACTGCAATATTCTTCTTAATTGCCTAC	
apc053		MCP-AID*mono Fragment 1 for EMY backbone
apc064	GGTGACCAAGAGATAAACCATGTAACACGATAACACC	
apc065	CGTGTTACATGGTTTATCTCTTGGTCACCATGCTATG	MCP-AID*mono Fragment 2 for EMY backbone
apc066	AGCTTTGAAGGTTCTACCATGATTTTCGACGAAGGTATTC	
apc067	GTCGAAAATCATGGTAGAACCTTCAAAGCTTGGG	MCP-AID*mono Fragment 3 for EMY backbone
apc068	GAGCTGATACCGCTCGGTC	
apc053		AID731Δ Fragment 1 for EMY backbone
apc069	GGTACAAAAATTCACTTCTATTCATTAATAGTGAATCCATAGAG	
apc070	GGATTCACTATTAATGAATAGAAGTGAATTTTTGTACCAATTTAAGAACGTG	AID731Δ Fragment 2 for EMY backbone
apc071	ACGAGAAAAGGAAGTTGCTGAGTCGCATCTTTTCACTACGTAACATAGATAAG	
apc072	TGCGACTCAGCAACTTCCTTTTCTCGTGATTTCGGTTACTTAAGAAATAAGAACG	AID731Δ Fragment 3 for EMY backbone

apc073	CAGCTACTAGTCTGGCACAATCAGAGCATGGTGACCAAGAG	
apc074	GCTCTGATTGTGCCAGACTAGTAGCTGATTTCTTACGTGGTAAC	AID731Δ Fragment 4 for EMY backbone
apc075	CAGCAGTAAAAGTAATCTTCGAAAGTCATTATTGCAATCTGAAC	
apc076	GCAATAATGACTTTCGAAGATTACTTTTACTGCTGGAATAC	AID731Δ Fragment 5 for EMY backbone
apc054		
apc053		MCP-altcodon_AID*Δ Fragment 1 (MCP) for EMY backbone
apc077	GGCTGCGGCCACTTTTCTC	
apc078	CTAAGAAAAAGAGAAAAGTGGCCGCAGCC	MCP-altcodon_AID*Δ Fragment 2 (human or alt. yeast codon optimized AID*Δ) for EMY backbone
apc068		
apc079	GAATGGTTAAGTAGCCAGGTGTCCATCGTGCCTAATCCAAGGAGGTTTAC	MCPz-AID*Δ Fragment 1 (pGal2) for HR1 backbone
apc080	GGCATCTCGAGAGACATTATGAAAGAATTATTTTTTTTATTATGTTAATCTTGTG	
apc081	AAAATAATTCTTTCATAATGTCTCTCGAGATGCCCAAAAAG	MCPz-AID*Δ Fragment 2 (MCPz) for HR1 backbone
apc082	CACCTCCAGATCCACCTCCTCCGTAGATGCCGGAGTTTGCTG	
apc083	GGAGGAGGTGGATCTGGAGGTGGAGGCTCTATGGATTCACTATTAATGAATAG	MCPz-AID*Δ Fragment 3 (AID*Δ) for HR1 backbone
apc084	CTTCAGCAACCGTCCTTTTATGTCCTGAATGCATCACG	
apc085	CATTCAGGACATAAAAGGACGGTTGCTGAAGAAAAAG	MCPz-AID*Δ Fragment 4 (Tvma2) for HR1 backbone
apc086	TCGACACAGGTCGCTTGGTCCTGAGGTGTGTTCCTTGATCTTTTTC	
apc079		MCP-AID*Δ-RFA3 Fragment 1 (Pgal2-MCP-AID*Δ) for HR1 backbone
apc087	CTGAACTTCCGCCGCTAGAACCTCCTGAAGAACCACCAGATTATGTCCTGAATGCATCACGTAAATC	
apc088	GAGGTTCTAGCGGCGGAAGTTCAGGTGGATCTTCTGGTGGATCCATGGCCAGCGAAACACCAAG	MCP-AID*Δ-RFA3 Fragment 2 (RFA3) for HR1 backbone
apc089	GCAACCGTCCTTCTAGTATATTTCTGGGTATTTCTTACATAG	
apc090	ACCCAGAAATATACTAGAAGGACGGTTGCTGAAGAAAAAG	MCP-AID*Δ-RFA3 Fragment 3 (Tvma2) for HR1 backbone
apc086		

Initial gRNA scaffolds (No MS2, M13, Mtx2, M4)

apc091	GAGCCAGTGAGTTGATTGGAAGACCTGGATCCTCTTTGAAAAGATAATGTATGATTATGCTT	pSNR52 for pY120 backbone
apc092	AATTCCGTCAGCCAGGGTCTCGATCATTTATCTTTCACTG	
apc093	GATAAATGATCGAGACCCTGGCTGACGGAATTTATGCC	Blank gap for pY120 backbone
apc094	CTAGCTCTGAAACTGAGACCGAGAAAACTCACCG	
apc095	GAGTGAGCTGATACCGCTCGAAGACGGATCCAGACATAAAAAACAAAAAAAGCACCG	Nested PCR M13 for pY120 backbone
apc096	GTTTCAGAGCTAGGCCAACATGAGGATCACCCATGTCTGCAGGGCCTAGCAAG	forward primer 1
apc097	GAGTTTTCTCGGTCTCAGTTTCAGAGCTAGGCCAACATG	forward primer 2
apc098	GTTTTCTCGGTCTCAGTTTCAGAGCTAGAAATAGCAAGTTG	M4 or No Ms2 for pY120 backbone
apc095		
apc098		Mtx2 Fragment 1 (gRNA scaffold) for pY120 backbone

apc099	TCCCGCACCGACTCGGTGCCAC	
apc100<br>apc095	AAGTGGCACCGAGTCGGTG	Mtx2 Fragment 2 (Mtx2) for pY120 backbone

Remaining gRNA scaffolds (M1, M3, M14, M34, M1tx2, M3tx2, M13tx2, Mt, M13t)

apc101<br>apc102	GAGCCAGTGAGTTGATTGGAAG<br>AAGTTGATAACGGACTAGCCTTATTTC	Fragment 1 (pSNR52, blank gap, M1) for pY120 backbone
apc103<br>apc104	AGCAAGTTGAAATAAGGCTAGTCC<br>GAGCTGATACCGCTCGAAGACCTGGATCCAG	Fragment 2 (No MS2 or M4) for pY120 backbone<br>[Makes M1 or M14]
apc101<br>apc102		Fragment 1 (pSNR52, blank gap, No MS2) for pY120 backbone
apc103<br>apc104		Fragment 2 (M3) for pY120 backbone<br>[Makes M3]
apc101<br>apc105<br>apc106	 AACAAAAAAAGCACATGGGTGATCCTCATGTGCGCGCACCGACTCGGTGCCAC<br>GCTGATACCGCTCGAAGACCTGCAGAGACATAAAAAACAAAAAAAGCACATGGGTGATCC	pSNR52-blank gap-[Mt or M13t] Nested PCR for pY120g backbone, forward primer<br>reverse primer 1<br>reverse primer 2
apc101<br>apc107	 TCCCGCACCGACTCGGTGCCAC	Fragment 1 (pSNR52, blank gap, M1 or M3 or M1,3) for pY120 backbone
apc108<br>apc104	AAGTGGCACCGAGTCGGTG	Fragment 2 (Mtx2) for pY120 backbone<br>[Makes M1tx2, M3tx2, or M13tx2]
apc101<br>apc109	 CCTCGGTGCCACTTGGCCCTGCAGACATGGGTGATCCTCATGTTGGCCAAGTTGATAACGGACTAGCC	Fragment 1 (pSNR52, blank gap, M3) for pY120 backbone
apc110<br>apc104	GCAGGGCCAAGTGGCACCGAGGCCAAC	Fragment 2 (M4) for pY120 backbone<br>[Makes M34]

gRNA spacers

apc111<br>apc112	TGATCCGGCGTCGAAGCCTGTAAAG<br>AAACCTTTACAGGCTTCGACGCCGG	Anneal for NT1
apc113<br>apc114	TGATCGGCGAGGGCGATGCCACCTA<br>AAACTAGGTGGCATCGCCCTCGCCG	Anneal for wtGFP t74L
apc115<br>apc116	TGATCCCGGCAAGCTGCCCGTGCCC<br>AAACGGGCACGGGCAGCTTGCCGGG	Anneal for wtGFP t22L
apc117<br>apc118	TGATCGTAGCTGAAGGTGGTCACGA<br>AAACTCGTGACCACCTTCAGCTACG	Anneal for wtGFP 18L
apc119<br>apc120	TGATCGCACTGCACGCCGTAGCTGA<br>AAACTCAGCTACGGCGTGCAGTGCG	Anneal for wtGFP 6L

apc121<br>apc122	TGATCGTGGTCACGAGGGTGGGCCA<br>AAACTGGCCCACCCTCGTGACCACG	Anneal for wtGFP 28L
apc123<br>apc124	TGATCGTCGTGCTGCTTCATGTGGT<br>AAACACCACATGAAGCAGCACGACG	Anneal for wtGFP 29R
apc125<br>apc126	TGATCGGGCACGGGCAGCTTGCCGG<br>AAACCCGGCAAGCTGCCCGTGCCCG	Anneal for wtGFP 48L
apc127<br>apc128	TGATCGACGTAGCCTTCGGGCATGG<br>AAACCCATGCCCGAAGGCTACGTCG	Anneal for wtGFP 62R
apc129<br>apc130	TGATCCTTCAGGGTCAGCTTGCCGT<br>AAACACGGCAAGCTGACCCTGAAGG	Anneal for wtGFP 81L
apc131<br>apc132	TGATCTGAAGAAGATGGTGCGCTCC<br>AAACGGAGCGCACCATCTTCTTCAG	Anneal for wtGFP 84R
apc153<br>apc154	TGATCTTCAAGTCCGCCATGCCCGA<br>AAACTCGGGCATGGCGGACTTGAAG	Anneal for wtGFP c78R

3x gRNA-tRNA cassettes

apc133<br>apc134	GCGTTGGCCGATTCATTAATG<br>CTCTGAAACTGAGACCGAAGGAGAAAACTCACCGAGG	gRNA-tRNA Fragment 1 for pUC backbone (used for Mtx2 and M13)
apc135<br>apc136	CTCCTTCGGTCTCAGTTTCAGAGCTAGAAATAGCAAG<br>AACCACTTGCGCTTGTTTGGGAACACGAGCGACATGG	Mtx2 gRNA-tRNA Fragment 2 (Mtx2) for pUC backbone
apc137<br>apc138	GTTCCCAAACAAGCGCAAGTGGTTTAGTGGTAAAATC<br>GGCCTCTTCGCTATTACGCC	Mtx2 gRNA-tRNA Fragment 3 (tRNA-Gly) for pUC backbone
apc139<br>apc140	CTCCTTCGGTCTCAGTTTCAGAGCTAGAAATAGCAAG<br>CACTTGCGCTTGTTTGCACCGACTCGGTGCCAC	M13 gRNA-tRNA Fragment 2 (M13) for pUC backbone
apc141<br>apc142	GTCGGTGCAAACAAGCGCAAGTGGTTTAGTGG<br>GGCCTCTTCGCTATTACGCC	M13 gRNA-tRNA Fragment 3 (tRNA-Gly) for pUC backbone
apc143<br>apc144	AAAGGTCTCATGATCCTCTTAAGTTGTTCATTTGCGTTTCAGAGCTAGAAATAGCAAGTTG<br>AAAGGTCTCAATGAAACTCTCTGCGCAAGCCCGGAATCG	mtx2 VH4420 3x cassette Fragment 1 for pY120 backbone
apc145<br>apc146	AAAGGTCTCATCATGGGCCTCCCGTTTCAGAGCTAGAAATAGCAAGTTG<br>AAAGGTCTCAAAACGAGAAAGGACTGGAGTGGGTTGCGCAAGCCCGGAATCG	mtx2 VH4420 3x cassette Fragment 2 for pY120 backbone
apc147<br>apc148	AAAGGTCTCATGATCGTCACTAAAAGTGAATCCAGGTTTCAGAGCTAGGCCAACATG<br>AAAGGTCTCAGAAACATATTATGCGCAAGCCCGGAATCG	m13 VH4420 3x cassette Fragment 1 for pY120 backbone
apc149<br>apc150	AAAGGTCTCATTTCATAATTATAGTTTCAGAGCTAGGCCAACATG<br>AAAGGTCTCAAAACGTACAGTAATAGATACCCATTGCGCAAGCCCGGAATCG	m13 VH4420 3x cassette Fragment 2 for pY120 backbone

Supplemental Table S4. gRNA scaffold sequences

gRNA variant	Annotated Sequence: modifications are highlighted, MS2 loops are bolded and underlined
No MS2	GTTTCAGAGCTAGAAATAGCAAGTTGAAATAAGGCTAGTCCGTTATCAACTTGAAAAAGTGGCACCGAGTCGGTGC
M13	GTTTCAGAGCTAGGCCA**ACATGAGGATCACCCATGT**CTGCAGGGCCTAGCAAGTTGAAATAAGGCTAGTCCGTTATCAACTTGGCCA**ACATGAGGATCACCCATGT**CTGCAGGGCCAAGTGGCACCGAGTCGGTGC
Mtx2	GTTTCAGAGCTAGAAATAGCAAGTTGAAATAAGGCTAGTCCGTTATCAACTTGAAAAAGTGGCACCGAGTCGGTGCGGGAGC**ACATGAGGATCACCCATGT**GCCACGAGCG**ACATGAGGATCACCCATGT**CGCTCGTGTTCCC
Mt	GTTTCAGAGCTAGAAATAGCAAGTTGAAATAAGGCTAGTCCGTTATCAACTTGAAAAAGTGGCACCGAGTCGGTGCGCGC**ACATGAGGATCACCCATGT**GC
M1	GTTTCAGAGCTAGGCCA**ACATGAGGATCACCCATGT**CTGCAGGGCCTAGCAAGTTGAAATAAGGCTAGTCCGTTATCAACTTGAAAAAGTGGCACCGAGTCGGTGC
M3	GTTTCAGAGCTAGAAATAGCAAGTTGAAATAAGGCTAGTCCGTTATCAACTTGGCCA**ACATGAGGATCACCCATGT**CTGCAGGGCCAAGTGGCACCGAGTCGGTGC
M4	GTTTCAGAGCTAGAAATAGCAAGTTGAAATAAGGCTAGTCCGTTATCAACTTGAAAAAGTGGCACCGAGGCCA**ACATGAGGATCACCCATGT**CTGCAGGGCCTCGGTGC
M14	GTTTCAGAGCTAGGCCA**ACATGAGGATCACCCATGT**CTGCAGGGCCTAGCAAGTTGAAATAAGGCTAGTCCGTTATCAACTTGAAAAAGTGGCACCGAGGCCA**ACATGAGGATCACCCATGT**CTGCAGGGCCTCGGTGC
M34	GTTTCAGAGCTAGAAATAGCAAGTTGAAATAAGGCTAGTCCGTTATCAACTTGGCCA**ACATGAGGATCACCCATGT**CTGCAGGGCCAAGTGGCACCGAGGCCA**ACATGAGGATCACCCATGT**CTGCAGGGCCTCGGTGC
M1tx2	GTTTCAGAGCTAGGCCA**ACATGAGGATCACCCATGT**CTGCAGGGCCTAGCAAGTTGAAATAAGGCTAGTCCGTTATCAACTTGAAAAAGTGGCACCGAGTCGGTGCGGGAGC**ACATGAGGATCACCCATGT**GCCACGAGCG**ACATGAGGATCACCCATGT**CGCTCGTGTTCCC
M3tx2	GTTTCAGAGCTAGAAATAGCAAGTTGAAATAAGGCTAGTCCGTTATCAACTTGGCCA**ACATGAGGATCACCCATGT**CTGCAGGGCCAAGTGGCACCGAGTCGGTGCGGGAGC**ACATGAGGATCACCCATGT**GCCACGAGCG**ACATGAGGATCACCCATGT**CGCTCGTGTTCCC
M13tx2	GTTTCAGAGCTAGGCCA**ACATGAGGATCACCCATGT**CTGCAGGGCCTAGCAAGTTGAAATAAGGCTAGTCCGTTATCAACTTGGCCA**ACATGAGGATCACCCATGT**CTGCAGGGCCAAGTGGCACCGAGTCGGTGCGGGAGC**ACATGAGGATCACCCATGT**GCCACGAGCG**ACATGAGGATCACCCATGT**CGCTCGTGTTCCC
M13t	GTTTCAGAGCTAGGCCA**ACATGAGGATCACCCATGT**CTGCAGGGCCTAGCAAGTTGAAATAAGGCTAGTCCGTTATCAACTTGGCCA**ACATGAGGATCACCCATGT**CTGCAGGGCCAAGTGGCACCGAGTCGGTGCGCGC**ACATGAGGATCACCCATGT**GC

Supplemental Table S5. gRNA spacer sequences

gRNA Spacer Name	Sequence
18L	GTAGCTGAAGGTGGTCACGA
t22L	CCGGCAAGCTGCCCGTGCCC
t74L	GGCGAGGGCGATGCCACCTA
NT1	CGGCGTCGAAGCCTGTAAAG

81L	CTTCAGGGTCAGCTTGCCGT
48L	GGGCACGGGCAGCTTGCCGG
28L	GTGGTCACGAGGGTGGGCCA
6L	GCACTGCACGCCGTAGCTGA
29R	GTCGTGCTGCTTCATGTGGT
62R	GACGTAGCCTTCGGGCATGG
84R	TGAAGAAGATGGTGCGCTCC
t78R	TTCAAGTCCGCCATGCCCGA
Mtx2, VHCDR1	GAGAGTTTCATGGGCCTCCC
Mtx2, VHCDR2	ACCCACTCCAGTCCTTTCTC
Mtx2, VHCDR3	CTCTTAAGTTGTTCATTTGC
M13, VHCDR1	GTCACTAAAAGTGAATCCAG
M13, VHCDR2	TAATATGTTTCATAATTATA
M13, VHCDR3	ATGGGTATCTATTACTGTAC
